# Evidence for the Circulation and Inter-Hemispheric Movement of the H14 Subtype Influenza A Virus

**DOI:** 10.1371/journal.pone.0059216

**Published:** 2013-03-28

**Authors:** Anthony C. Fries, Jacqueline M. Nolting, Angela Danner, Robert G. Webster, Andrew S. Bowman, Scott Krauss, Richard D. Slemons

**Affiliations:** 1 The Ohio State University, Department of Veterinary Preventive Medicine, Columbus, Ohio, United States of America; 2 St. Jude Children’s Research Hospital, Department of Infectious Diseases, Memphis, Tennessee, United States of America; National Institute for Viral Disease Control and Prevention, CDC, China

## Abstract

Three H14 influenza A virus (IAV) isolates recovered in 2010 during routine virus surveillance along the Mississippi Migratory Bird Flyway in Wisconsin, U.S.A. raised questions about the natural history of these rare viruses. These were the first H14 IAV isolates recovered in the Western Hemisphere and the only H14 IAV isolates recovered since the original four isolates in 1982 in Asia. Full length genomic sequencing of the 2010 H14 isolates demonstrated the hemagglutinin (HA) gene from the 1982 and 2010 H14 isolates showed 89.6% nucleotide and 95.6% amino acid similarity and phylogenetic analysis of these viruses placed them with strong support within the H14 subtype lineage. The level of genomic divergence observed between the 1982 and 2010 viruses provides evidence that the H14 HA segment was circulating undetected in hosts and was not maintained in environmental stasis. Further, the evolutionary relationship observed between 1982 H14 and the closely related H4 subtype HA segments were similar to contemporary comparisons suggesting limited adaptive divergence between these sister subtypes. The nonstructural (NS) segment of one 2010 isolate was placed in a NS clade isolated infrequently over the last several decades that includes the NS segment from a previously reported 1982 H14 isolate indicating the existence of an unidentified pool of genomic diversity. An additional neuraminidase reassortment event indicated a recent inter-hemispheric gene flow from Asia into the center of North America. These results demonstrate temporal and spatial gaps in the understanding of IAV natural history. Additionally, the reassortment history of these viruses raises concern for the inter-continental spread of IAVs and the efficacy of current IAV surveillance efforts in detecting genomic diversity of viruses circulating in wild birds.

## Introduction

All novel influenza A virus (IAV) strains resulting in global pandemics since the 20^th^ century have contained genetic elements from avian-origin IAV lineages [1], [2]. Therefore, detection and characterization of avian-origin IAVs in their natural host species is critical to elucidating the ecology and ancestral origins of IAVs and ultimately protecting public health. It is well established that wild birds in the order Anseriformes and Chardriiformes are natural reservoirs for antigenically and genetically diverse populations of influenza A viruses, including 16 of the 17 known influenza A virus hemagglutinin (HA) subtypes and 9 of the 10 known neuraminidase (NA) subtypes [3]–[7]. Many of these subtypes have strong host species preferences or even appear to be host species specific [7], [8]. As negative sense RNA viruses with segmented genomes, IAVs undergo frequent genomic reassortment resulting in genetically diverse and transient genomic constellations in wild bird populations [9], [10].

Understanding the ecology of IAVs is complicated by the ability of migratory birds to transport IAVs over large geographic expanses facilitating the movement of viruses and their genomic segments worldwide [11]. Interestingly, recent studies suggest that some IAV strains are preferentially maintained within individual migratory flyways and may be endemic to certain regions of the world [12], [13]. Additionally problematic, IAVs may persist in environmental stasis for long periods of time, undetected by surveillance focused on wild birds, until a time when agent, host, or environmental factors allow for reemergence in susceptible hosts within a region [14]. The rapid spread and lack of detection of many IAVs is particularly concerning when one considers the highly pathogenic strains of IAV which are lethal to domestic poultry and humans.

In the last 40 years tens of thousands of IAV isolates have been recovered around the world, but prior to 2010 only four isolates have possessed the H14 HA subtype [4]. During the fall waterfowl migration of 2010, routine virus surveillance efforts in the Mississippi Migratory Bird Flyway resulted in the recovery of three H14 influenza A virus isolates from sea ducks: two from long-tailed ducks (*Clangula hyemalis*) and one from a white-winged scoter (*Melanitta fusca*) [15]. These are the only known isolations of H14 subtypes in the 28 years since the original isolation of H14 IAVs from mallards (*Anas platyrhynchos*) and a herring gull (*Larus argentatus*) sampled in Central Asia along the northern shore of the Caspian Sea in 1982 [4]. The absence of H14 IAVs since 1982 raises questions as to how and where these viruses were maintained. Given the absence of detection at a time of globally increased influenza A virus surveillance, an investigation into the evolution of these unique viruses was warranted.

The objective of this investigation was to compare the genetic relationship of H14 IAV isolates recovered in 2010 to the original H14 IAV isolates recovered in 1982 to determine if H14 IAVs are circulating in a host species and/or an ecological niche that is not included in current surveillance efforts or whether the viruses have been held in stasis in the environment during the 28 year gap between recoveries and only recently spilled over into a host species. We also examine the eight genomic segments of each of these three contemporary H14 IAV isolates to determine if the geographic origin and temporal patterns of segment diversity provide any clues as to where these unique viruses were maintained when they were introduced to the western hemisphere. The phylogenetic analyses of these 2010 H14 IAV isolates indicate a potentially under sampled pool of IAV genetic diversity in which reassortment with contemporary wild bird IAVs is ongoing and interhemispheric movement of genomic segments is undetected.

## Materials and Methods

### Viral RNA Extraction

The influenza viruses A/LTDU/WI/10OS3912/2010(H14N6), A/WWSC/WI/10OS3922/2010(H14N4,8), and A/LTDU/WI/10OS4225/2010(H14N6) were isolated in embryonated chicken eggs as previously described [15]. All wild bird viral RNAs were obtained from first egg passage allantoic fluid using RNA isolation kit RNeasy® (Qiagen, Inc., Valencia, CA) following the manufacturer’s recommended protocol. PCR reactions were performed using the OneStep RT-PCR kit® (Qiagen, Inc., Valencia, CA) and when necessary the products were extracted using the Qiaquick Gel Extraction Kit® (Qiagen, Inc., Valencia, CA) following the manufacturer’s recommended protocol.

### Sequencing

The entire length of each genomic segment was sequenced at The Hartwell Center for Bioinformatics and Biotechnology at St. Jude Children’s Research Hospital on Applied Biosystems 3730XL DNA analyzers (Applied Biosystems, Foster City, CA) using BigDye Terminator (v. 3.1) chemistry (Applied Biosystems, Foster City, CA). The large number of RNA templates (10^∧^6–10^∧^9 infectious particles per mL) for cDNA synthesis in addition to Sanger sequencing with forward and reverse primers on overlapping fragments impeded RT-PCR infidelities in sequence reads. Sequencing of the entire untranslated sequence (Uni12) and 2–3 nucleotides of the gene specific untranslated sequence were mandated by the primers used during PCR amplification [4], [16]. We designed additional primers to validate questionable regions and these are available from the authors upon request. The sequences for all eight segments of the three contemporary H14 influenza A virus isolates have been deposited in GenBank with the accession numbers: JN696314-HN696316; KC110594-KC110614.

### Phylogenetic Analysis

Only the open reading frames of each segment were used for sequence comparison and phylogenetic analyses. Gene sequences were aligned using Geneious Pro (5.6.6) (Biomatters Ltd. Auckland, New Zealand) and Bioedit Sequence Alignment Editor (v. 5.0.6) (Ibis Biosciences Carlsbad, CA). Genetic sequences for segments available for the four H14 viruses isolated from birds in 1982 were obtained from the online public databases (Table S1). In addition, the Basic Local Alignment Search Tool (BLAST) from the National Center for Biotechnology Information [17] was used to assess the relationship of each gene segment from the 2010 H14 isolates with those sequences available on public databases.

To compare the level of genetic divergence within other HA subtypes over a similar time period as the H14 comparison, we obtained sequences of other HA subtypes by querying the Influenza Research Database [18], and restricting results by time period (Past: ≤1989; Present ≥2009) (Table S1). The availability of sequences for both the past and present classifications determined the range of years selected for each HA subtype. Narrower time periods were chosen from subtypes that were overrepresented in the database. MEGA5 [19] was used to compare genomic sequences of the isolates from 2010 to other isolates obtained from GenBank. Maximum Likelihood (ML) phylogenetic trees were constructed using RAxML [20]. We used a GAMMAGTR nucleotide model for nucleotide sequence trees and a BLOSUM62 amino acid model for protein trees. The highest likelihood tree for each segment was then run with 250 standard bootstrap replicates and the consensus trees with bootstrap support values and midpoint rooting are presented in the figures.

### Ethics Statement

All procedures involving animals were approved by The Ohio State University Institutional Animal Care and Use Committee (2007A0148-R1). Animals were collected on public property under U.S. Fish and Wildlife, U.S. Department of Interior permit MB219513-1. Sampling activities were coordinated with the Wisconsin Department of Natural Resources.

## Results and Discussion

### Sequence Analysis

The nucleotide sizes of the eight sequenced fragments from the 2010 H14 IAV isolates were as follows: 2341 bp (PB2), 2341 bp (PB1), 2233 bp (PA), 1749 bp (HA), 1565 bp (NP), 1460 bp (NA), 1027 bp (M), 890 bp (NS). There was one polymorphic, synonymous substitution within the 2010 H14 HA genomic segments indicative of a single virus introduction event at the geographic sampling location in Wisconsin, U.S.A. The presence of a lysine at the cleavage site of HA_0_ confirms the original finding in the H14 isolates from 1982 while all other HA subtypes contain arginine at the cleavage site [4]. Within the 2010 H14 viruses, five of the eight genomic segments (PB1, PB2, PA, M and NP) were typical of genetic sequences available from contemporary avian influenza A viruses circulating in North America (Table 1). However, the HA, NA and NS segments showed unique phylogenetic patterns and are discussed in further detail.

**Table 1:** The closest publically available influenza A virus segment sequences based on percent nucleotide similarity.

	A/LTDU/WI/10OS3912/2010/H14N6	A/WWSC/WI/10OS3922/2010/H14N8	A/LTDU/WI/10OS4225/2010/H14N6
PB2	A/MALL/MN/Sg-00462/2008/H6N1	A/MALL/MN/Sg-00462/2008/H6N1	A/MALL/MN/Sg-00462/2008/H6N1
	CY042385 (98.9%)	CY042385 (98.9%)	CY042385 (98.9%)
PB1	A/NOPI/MN/Sg-00227/2006/H1N1	A/NOPI/MN/Sg-00227/2006/H1N1	A/NOPI/MN/Sg-00227/2006/H1N1
	CY042227 (99.2%)	CY042227 (99.1%)	CY042227 (99.1%)
PA	A/MALL/AK/5/2007/H4N6	A/MALL/AK/5/2007/H4N6	A/MALL/AK/5/2007/H4N6
	CY039857 (98.9%	CY039857 (99.1%)	CY039857 (99.1%)
HA	**A/MALL/AST/263/1982/H14N5**	**A/MALL/AST/263/1982/H14N5**	**A/MALL/AST/263/1982/H14N5**
	**CY014604 (89.3%)**	**CY014604 (89.3%)**	**CY014604 (89.4%)**
NP	A/BWTE/GT/CIP049-05/2010/H3N8	A/Chicken/OH/494832/2007/H2N3	A/Chicken/OH/494832/2007/H2N3
	CY096657 (99.1%)	JF327339 (99.0%)	JF327339 (98.9%)
NA	**A/Avian/JP/8KI0185/2008/H4N6**	A/Environment/MD/1175/2005/H2N3	**A/Avian/JP/8KI0185/2008/H4N6**
	**CY079221 (98.8%)**	CY022727 (98.7%)	**CY079221 (98.7%)**
M	A/MALL/ALB/206/1996/H6N8	A/MALL/ALB/206/1996/H6N8	A/MALL/ALB/206/1996/H6N8
	CY004267 (99.1%)	CY004267 (99.1%)	CY004267 (99.1%)
NS	A/AGWT/AK/3/2007/H3N8	A/AGWT/AK/3/2007/H3N8	**A/MALL/AST/244/1982/H14N6**
	CY038390 (98.7%)	CY038390 (98.8%)	**CY005396 (95.3%)**

Percent nucleotide similarity was determined by NCBI BLAST analysis. Each genomic segment of the 2010 H14 subtype isolates is shown with the most similar accessioned sequences available on public databases. The GenBank accession number and the percentage of identical nucleotide sites shared with the 2010 H14 subtype isolate segments are indicated below each representative match.

+ Sequences in bold represent segments of Eurasian origin.

### Hemagglutinin Segment

**Figure 1:**
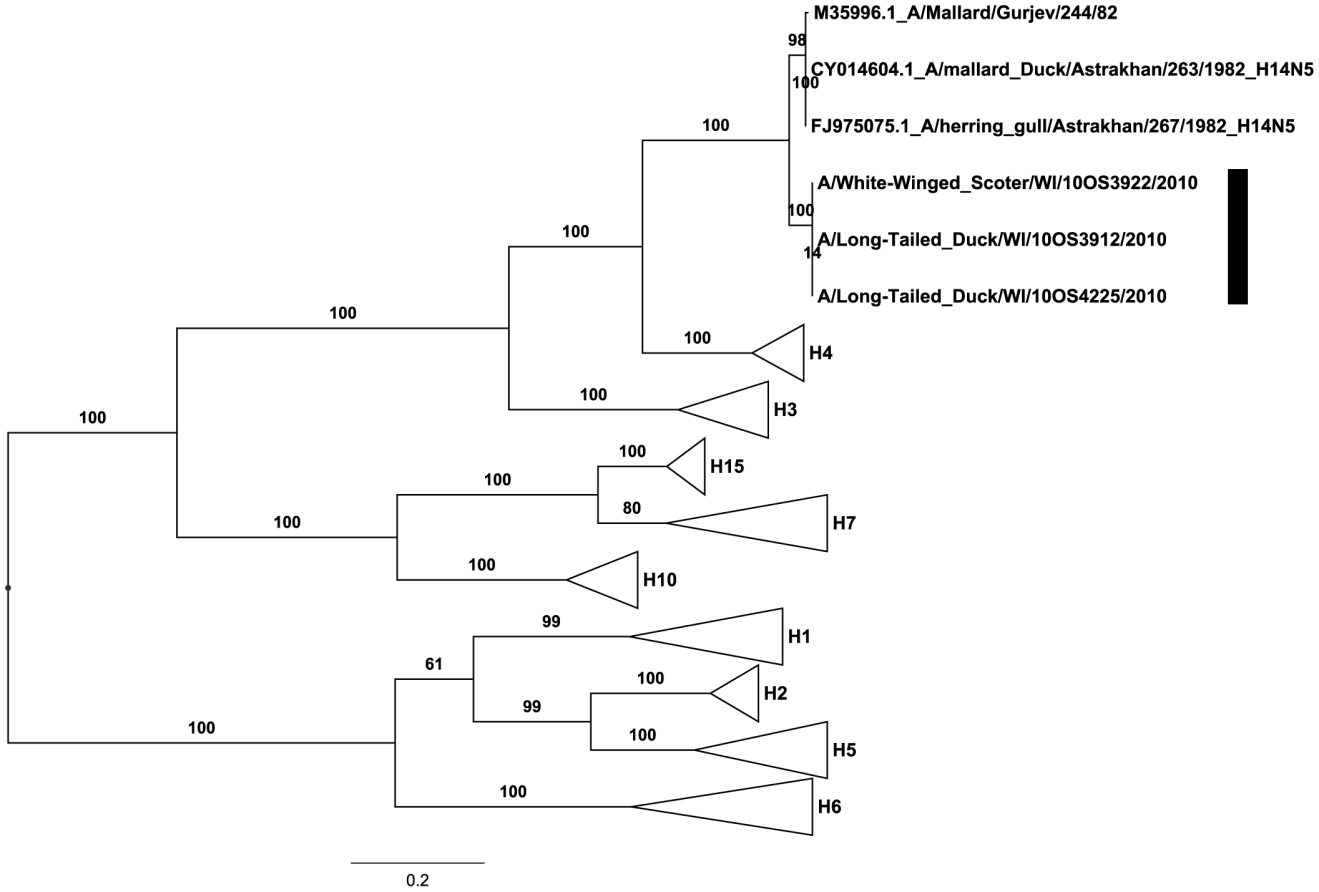
Phylogeny of the hemagglutinin segments of representative influenza A virus subtypes. Phylogeny of the influenza A virus HA genes from available sequences on the NCBI GenBank database (Table S1). Translated amino acid alignments and maximum likelihood (ML) trees were constructed using the open reading frame of the HA_0_ of each subtype. We assumed a BLOSUM62 amino acid model. This tree represents the consensus tree of 250 standard bootstrap iterations represented as a percentage of support for branch labels.

The BLAST results showed the HA segments of the 2010 viruses were most similar to HA segments sequenced from the H14 viruses recovered in 1982 (Table 1). The 1982 and 2010 HA genomic segments were 89.6% (1526/1704) similar in nucleotide sequence with 178 fixed differences. Of these 178 fixed differences, 25 were non-synonymous substitutions which resulted in a 95.6% (543/568) amino acid similarity. Of the 25 amino acid substitutions, 23 occurred within the HA_1_ segment while only 2 were observed in the HA_2_ segment consistent with the typical conservative evolution of HA_2_. The HA_0_ segments are firmly supported in the H14 clade (Figure 1) confirming the findings of Nolting et al. 2012 [15] based on partial sequences of the HA segment. The mean genetic distance based on amino acid sequence between the 1982 and 2010 viruses within the H14 clade was 0.048 (SE = 0.009) amino acid substitutions per site. This result is within the range of similar past vs. present comparisons within other subtypes (H4−>0.030; H6−>0.165) (Table 2). The comparison between amino acid divergence in pre-1985 H4 viruses and 1982 H14 viruses showed a difference of 0.261 (SE = 0.021) amino acid substitutions per site (Table 3). Interestingly, the same comparison done with viruses of each subtype in post-2009 viruses showed a 0.263 (SE = 0.021) amino acid substitutions per site relationship even with the 0.048 divergence observed within the H14 lineage. This pairwise relationship is similar to that seen in other genetically related subtypes (Table 3).

**Table 2:** Amino acid divergence observed within subtypes over time.

Subtype Comparison	Mean Distance	Standard Error
H1	0.090	0.006
H2	0.057	0.007
H3	0.065	0.006
H4	0.030	0.004
H5	0.122	0.011
H6	0.165	0.015
H7	0.090	0.008
H10	0.058	0.007
H14	0.048	0.009
H15	0.060	0.009

Estimates of evolutionary divergence for representative amino acid sequences between influenza A virus HA subtype lineages from the past (≤1989) and present (≥2009). Distances represent the average pairwise amino acid substitutions per site over all sequence pairs between groups. Standard error estimates were obtained by using a bootstrap procedure (1000 replicates). A total of 592 positions (non-ambiguous) over 604 sequences were used. A Poisson correction model was implemented. All analyses were conducted in MEGA5.

**Table 3:** Amino acid divergence observed between sister subtypes over time.

Subtype Comparison	Past (<1989)	Present (>2009)
H4 vs. H14	0.261 (0.021)	0.263 (0.021)
H2 vs. H5	0.276 (0.020)	0.288 (0.021)
H7 vs. H15	0.235 (0.019)	0.226 (0.019)

Estimates of evolutionary divergence for representative amino acid sequences between influenza A virus HA sister subtype lineages from the past (<1989) and present (>2009). Distances represent the average pairwise amino acid substitutions per site over all sequence pairs between groups. Standard error estimates were obtained by using a bootstrap procedure (1000 replicates). A total of 592 positions (non-ambiguous) over 604 sequences were used. A Poisson correction model was implemented. All analyses were conducted in MEGA5.

Based on the HA amino acid sequence divergence of 0.048 amino acid substitutions per site between the original 1982 and 2010 isolates it is likely that the H14 subtype has circulated in a temporal or spatial niche that is currently under represented in wild bird surveillance efforts around the world. This divergence was similar to the level of divergence seen between comparable time frames within other HA subtype genomic segments (Table 2). Kawaoka et al. 1990 [4] suggested that the amount of divergence observed between the H4 and H14 sister subtypes was consistent with recent divergence of the subtypes. While this is likely the case, after 30 years of circulation we saw the same level of divergence which is similar to the stable amino acid divergence values observed in other sister subtype comparisons (Table 3). This suggests that while significant amino acid substitutions are occurring within the subtype lineages, the amount of divergence observed between subtype lineages over similar time frames seems to be consistent due to substitution saturation at sites under selection in HA_1_. This makes dating divergence times between H4 and H14 difficult [8], [21].

One possible explanation for why the H14 subtype is not routinely detected may be due to competitive exclusion provided by cross-protective antibodies by the more common H4 sister subtype [9], [22]. Cross protective antibodies against commonly circulating low pathogenic IAV subtypes in waterfowl are known to influence infectivity of high pathogenic H5N1 [22]. However, since the H4 and H14 subtypes are evolutionarily the two youngest subtypes to diverge from each other, we may expect a greater degree of sequence divergence if they are still occupying similar environmental niches [4], [8]. While the amount of sequence divergence within the H14 lineage has increased by a similar amount as other more commonly circulating genomic subtypes (Table 2), we see that the amount of adaptive divergence between the H4 and H14 virus since 1982 has not changed. The accumulated evidence indicates that H14 genomic segments are circulating in an under represented environmental niche and is only periodically detected in spillover hosts by current surveillance efforts. Interestingly, the relationship between the H7 and rare H15 sister subtypes exhibit similar evolutionary relationships to that observed in H4 and H14.

### Nonstructural Segment

**Figure 2:**
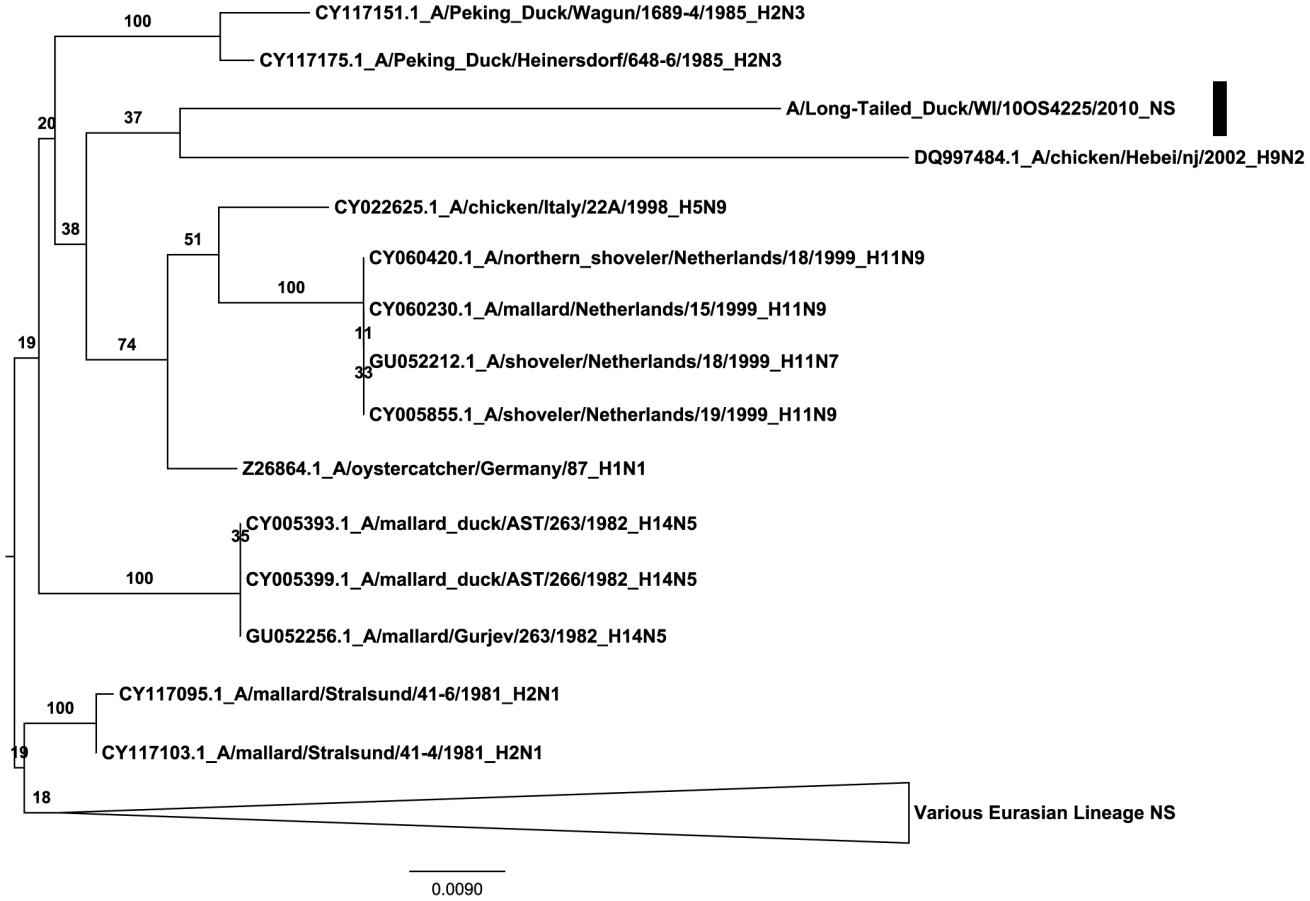
Phylogeny of nonstructural segments of influenza A viruses. Phylogeny of the influenza A virus NS gene segment from A/10OS4225/LTDU/2010 and the closest 250 sequences that were identified using the BLAST utility on NCBI (Table S2). A maximum likelihood (ML) consensus tree was constructed using the entire nucleotide open reading frame of the NS gene. We assumed a GAMMAGTR model with 250 standard bootstrap iterations represented as a percentage of support for branch labels.

The A/LTDU/WI/10OS4225/2010 nonstructural (NS) segment showed high similarity in a nucleotide BLAST analysis with the original A/MALL/AST/244/1982 NS segment showing 95.3% nucleotide similarity (Table 1). In addition, among the next 250 closest sequences available on GenBank all were of Eurasian origin (Table S2). A ML tree of these NS segments showed a poorly supported but suggestive clade in which the A/LTDU/WI/10OS4225/2010 NS segment is placed with older isolates including the 1982 H14 NS segments and exhibits a long branch length relative to other sequences in the NS tree (Figure 2). This tree topology indicates a large amount of undetected diversity similar to the HA segment and is indicative of circulation in a unique niche. This unusual amount of divergence is atypical of the diversity seen in allele B of the NS segment in waterfowl [23]. The phylogenetic relationship with the original A/MALL/AST/263/1982 is contradictory to suggestions that the NS segment shows the least amount of linkage to other internal segments of influenza A viruses [9].

### Neuraminidase Segment

**Figure 3:**
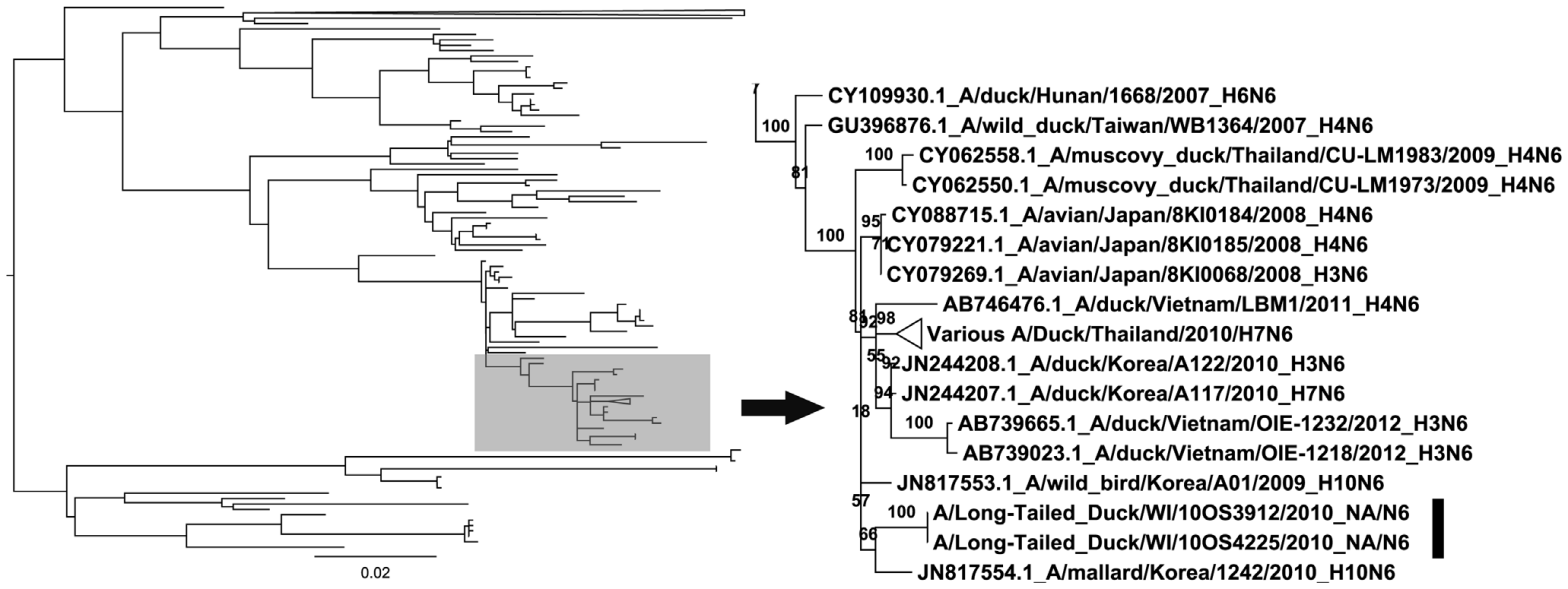
Phylogeny of neuraminidase subtype 6 segments of influenza A viruses. Phylogeny of the influenza A virus NA subtype 6 genetic segment from the A/10OS3912/LTDU/2010 and A/10OS4225/LTDU/2010 NA segments and the closest 250 sequences available on the NCBI GenBank database (Table S3). A maximum likelihood (ML) consensus tree was constructed using the entire nucleotide open reading frame of the NA gene. We assumed a GAMMAGTR model with 250 standard bootstrap iterations represented as a percentage of support for branch labels.

We constructed a NA subtype 6 segment tree based on the closest 250 viruses identified on GenBank by BLAST (Table S3). The NA segments of the 2010 H14 viruses from long-tailed ducks (3912 and 4225) were grouped in the NA ML tree with NA subtype 6 segments that were concurrently circulating in domestic and wild birds in Southeast Asia in 2010 (Figure 3). These segments showed 98.6% and 98.5% nucleotide and amino acid similarity with the closest available sequence on public databases, respectively.

While the NS and HA segments took several years to show up in North America, the neuraminidase sequences of the H14 viruses isolated in 2010 show a rapid transfer of Southeast Asian lineage N6 subtype into the Great Lakes region of North America. Further, the N8 reassortment event in A/WWSC/10OS3922/2010 supports a point source introduction of the H14 subtype and subsequent reassortment with co-circulating waterfowl IAVs in the region, consistent with the one synonymous substitution observed in the HA segments (Table 1). The N6 tree topology showed both the A/LTDU/WI/10OS4225/2010 and A/LTDU/WI/10OS3912/2010 NA segments were from N6 genomic diversity circulating in birds in Southeast Asia in 2010. Interestingly, even with the relatively recent movement of the N6 segment, the introduction of the 2010 H14 isolates in Wisconsin, U.S.A. still reassorted in 7 of the 8 segments of the A/WWSC/WI/10OS3922/2010 isolate. Notably, this inter-hemispheric movement of Eurasian lineage HA, NA and NS gene segments by either a migratory host or as an unintended consequence of human activities evaded current surveillance efforts aimed at detecting high pathogenic H5N1 in North American wild bird flyways [24]. The detection of H14 IAVs in the interior of North America is even more concerning considering that H14 IAVs, similar to H5 and H7 IAVs, can support a highly pathogenic phenotype resulting from the acquisition of a polybasic cleavage site [25].

### Unique Hosts

Type A influenza virus surveillance in Anseriformes is noticeably skewed towards dabbling ducks (genus *Anas*) comprising 39.5% of surveillance efforts while sea ducks comprise 0.7% of the over 200,000 avian surveillance samples listed on the Influenza Research Database [18]. However, sea ducks make up 28.0% of North American Anseriformes species which include eiders, scoters, mergansers, goldeneyes, long-tailed duck and harlequin ducks [26]. This disparity in sampling is likely a result of difficult access to sea ducks and a tendency for these species to stay well north of the major migratory movements of North American continental dabblers [26]. Sampling of some species of waterbirds is justifiably avoided due to documented low prevalence rates with the recovery of the most isolates and subtype diversity from more abundant and accessible birds such as mallards [27]. This finding of the unique genomic constellations of the contemporary H14 viruses from sea ducks should warrant further exploration into the role these species play in the ecology and epidemiology of influenza A viruses.

### Conclusion

This study showed that the H14 IAV isolates recovered in 2010 have diverged from the original 1982 H14 viruses in a manner consistent with continuous circulation in a host species. This 28 year gap in combination with the genetic divergence provides evidence for an ecological niche that is currently under represented in global surveillance efforts. The possibility of an overlooked ecological niche that maintains distinct lineages of influenza A viruses is further supported by the recent discovery of a new H17 and N10 subtype in bats [5], [28]. Other possible explanations for why the H14 subtype has not become established in commonly surveyed wild bird species include innate host species differences, cross-reactive antibodies generated against commonly circulating H4 viruses and circulation in a different host system. Further, the reassortment history of these three contemporary H14 IAV isolates suggests that intercontinental movement of genomic segments and formation of transient genomic constellations can occur relatively quickly and raises concerns about the efficacy of IAV surveillance efforts in wild birds to adequately capture influenza A virus genetic diversity.

## Supporting Information

Table S1 List of hemagglutinin segment sequences. List of the HA segments used in the sequence comparisons and phylogeny in this study. Sequences were selected based on the amount of sequences available for a given time frame from the past (≤1989) and present (≥2009).
(PDF)

Table S2 List of nonstructural segment sequences. List of the NS segments used in the phylogeny constructed in this study. Sequences were selected based on a BLAST analysis of the A/10OS4225/LTDU/2010 NS segment and subsequent identification of the closest 250 sequences available on GenBank. Accession numbers highlighted (Gray) represent sequences that were included in the rare lineage clade with the A/10OS4225/LTDU/2010 NS segment.
(PDF)

Table S3 List of neuraminidase segment sequences. List of the NA subtype 6 segments used in the phylogeny constructed in this study. Sequences were selected based on a BLAST analysis of the A/10OS3912/LTDU/2010 and A/10OS4225/LTDU/2010 NA segments and subsequent identification of the closest 250 sequences available on GenBank.
(PDF)
