# Supplementary material for: Evidence for the Circulation and Inter-Hemispheric Movement of the H14 Subtype Influenza A Virus
Source: PLoS One. 2013 Mar 28;8(3):e59216. doi: 10.1371/journal.pone.0059216 (PMC3610705; doi:10.1371/journal.pone.0059216)
Supplement: Table S1 — List of hemagglutinin segment sequences. List of the HA segments used in the sequence comparisons and phylogeny in this study. Sequences were selected based on the amount of sequences available for a given time frame from the past (≤1989) and present (≥2009). (PDF) [file pone.0059216.s001.pdf]

Accession_Number	Common_Name	Location	Year	Subtype	Time_Comparison
CY087760.1	Shearwater	Australia	1972	H10N8	Past
CY006030.1	Duck	Hong Kong	1976	H4N2	Past
CY005921.1	Northern Pintail	Alberta	1977	H10N7	Past
CY005993.1	Mallard	Alberta	1977	H10N7	Past
CY005968.1	Mallard	Alberta	1977	H4N1	Past
CY005948.1	Mallard	Alberta	1977	H4N3	Past
CY005944.1	Canvasback	Alberta	1977	H4N6	Past
CY005946.1	Redhead	Alberta	1977	H4N6	Past
CY005947.1	Blue-winged Teal	Alberta	1977	H4N6	Past
CY005951.1	Mallard	Alberta	1977	H4N6	Past
CY005950.1	Mallard	Alberta	1977	H4N8	Past
CY005952.1	Northern Pintail	Alberta	1977	H4N8	Past
CY005994.1	Blue-winged Teal	Alberta	1978	H10N3	Past
CY005538.1	Duck	Hong Kong	1978	H2N2	Past
CY116835.1	Mallard	New York	1978	H2N2	Past
CY116891.1	Duck	Hong Kong	1978	H2N2	Past
L11128.1	Duck	Hong Kong	1978	H2N2	Past
L11137.1	Mallard	New York	1978	H2N2	Past
CY117243.1	Black Duck	New Jersey	1978	H2N3	Past
CY117251.1	Mallard	New York	1978	H2N3	Past
CY120777.1	Blue-winged Teal	Alberta	1978	H2N3	Past
AB292785.1	Duck	Hong Kong	1978	H2N9	Past
CY005546.1	Duck	Hong Kong	1978	H2N9	Past
CY005953.1	Mallard	Alberta	1978	H4N2	Past
CY006027.1	Duck	Hong Kong	1978	H4N6	Past
CY005575.1	Duck	Hong Kong	1978	H5N2	Past
EF597251.1	Duck	Hong Kong	1978	H5N2	Past
AB542809.1	Duck	Hong Kong	1978	H5N3	Past
AF290443.1	Duck	Ho Chi Minh	1978	H5N3	Past
CY006028.1	Goose	Hong Kong	1978	H5N3	Past
EF597248.1	Duck	Hong Kong	1978	H5N3	Past
EF597249.1	Duck	Hong Kong	1978	H5N3	Past
EF597252.1	Goose	Hong Kong	1978	H5N3	Past
AB292412.1	Duck	Hong Kong	1979	H10N3	Past
CY014739.1	Mallard	Minnesota	1979	H10N7	Past
GU186624.1	Turkey	Minnesota	1979	H10N7	Past
AB292781.1	Duck	Hong Kong	1979	H10N9	Past
CY014619.1	Duck	Hong Kong	1979	H10N9	Past
GU052260.2	Shearwater	Australia	1979	H15N6	Past
CY006010.1	Wedge-tailed Shearwater	Western Australia	1979	H15N9	Past
CY077616.1	Australian Shelduck	Western Australia	1979	H15N9	Past
GQ247870.1	Shearwater	Australia	1979	H15N9	Past
L43917.1	Shearwater	West Australia	1979	H15N9	Past
AB292406.1	Mallard	Alberta	1979	H4N2	Past
CY005954.1	Mallard	Alberta	1979	H4N2	Past
CY005955.1	Blue-winged Teal	Alberta	1979	H4N2	Past

CY005956.1	Northern Pintail	Alberta	1979	H4N2	Past
CY014922.1	Blue-winged Teal	New York	1979	H4N2	Past
CY005672.1	Gray Teal	Australia	1979	H4N4	Past
CY045263.1	Gray Teal	Western Australia	1979	H4N4	Past
M25284.1	Gray Teal	Australia	1979	H4N4	Past
CY005679.1	Gray Teal	Australia	1979	H4N6	Past
CY031156.1	Gray Teal	Western Australia	1979	H4N6	Past
AF082039.1	Duck	Hong Kong	1979	H5N3	Past
EF597250.1	Duck	Hong Kong	1979	H5N3	Past
EU743301.1	Turkey	California	1979	H5N3	Past
GU052914.1	Northern Pintail	Alberta	1979	H7	Past
AF149295.1	African Starling	England	1979	H7N1	Past
AF202232.1	African Starling	England	1979	H7N1	Past
AF202235.1	Turkey	Israel	1979	H7N2	Past
AB269692.2	Turkey	Tennessee	1979	H7N3	Past
AF202245.1	Turkey	England	1979	H7N3	Past
CY107844.1	Chicken	Germany	1979	H7N7	Past
CY107860.1	Tern	Potsdam	1979	H7N7	Past
L43913.1	Goose	Leipzig	1979	H7N7	Past
L43914.1	Goose	Leipzig	1979	H7N7	Past
L43915.1	Goose	Leipzig	1979	H7N7	Past
U20459.1	Chicken	Leipzig	1979	H7N7	Past
AB271117.1	Duck	Hong Kong	1980	H10N1	Past
CY014627.1	Duck	Australia	1980	H1N1	Past
CY094927.1	Duck	Victoria	1980	H1N1	Past
EU742636.2	Turkey	Kansas	1980	H1N1	Past
L25071.1	Duck	Wisconsin	1980	H1N1	Past
L25072.1	Duck	Wisconsin	1980	H1N1	Past
CY117059.1	Mallard	Berlin	1980	H2N3	Past
CY117067.1	Mallard	Rugen	1980	H2N3	Past
AB292402.1	Duck	Hong Kong	1980	H3N1	Past
AB275283.2	Duck	Hokkaido	1980	H3N8	Past
CY005977.1	Northern Pintail	Alberta	1980	H3N8	Past
CY014633.1	Red-necked Stint	Australia	1980	H3N8	Past
CY028275.1	Red-necked Stint	Bunbury	1980	H3N8	Past
AB292404.1	Duck	Hong Kong	1980	H4N1	Past
CY014751.1	Turkey	Minnesota	1980	H4N2	Past
M25290.1	Turkey	Minnesota	1980	H4N2	Past
CY094919.1	Duck	Victoria	1980	H4N6	Past
CY014630.1	Red-necked Stint	Australia	1980	H4N8	Past
CY014723.1	Domestic Duck	Minnesota	1980	H4N8	Past
CY033153.1	Red-necked Stint	Western Australia	1980	H4N8	Past
CY014580.1	Mallard	Alberta	1980	H5N2	Past
CY021381.1	Turkey	Italy	1980	H5N2	Past
GQ247849.1	Turkey	Italy	1980	H5N2	Past
U79449.1	Duck	Michigan	1980	H5N2	Past
EF597253.1	Duck	Hong Kong	1980	H5N3	Past

AB294213.1	Duck	Hong Kong	1980	H6N2	Past
CY005691.1	Black Duck	Australia	1980	H6N5	Past
CY077660.1	Pacific Black Duck	Western Australia	1980	H6N5	Past
CY005881.1	Blue-winged Teal	Minnesota	1980	H6N6	Past
CY014764.1	Turkey	Minnesota	1980	H6N6	Past
CY014721.1	Pheasant	Minnesota	1980	H7N3	Past
CY014778.1	Turkey	Minnesota	1980	H7N3	Past
CY041839.2	Turkey	Minnesota	1980	H7N3	Past
AF202250.1	Macaw	England	1980	H7N7	Past
CY081275.1	Duck	Potsdam	1980	H7N7	Past
CY107850.1	Duck	Potsdam	1980	H7N7	Past
CY116741.1	Mallard	Rugen	1980	mixed	Past
AB289339.1	Swan	Shimane	1981	H10N6	Past
AF091310.1	Turkey	Minnesota	1981	H1N1	Past
CY005866.1	Pigeon	Minnesota	1981	H1N1	Past
CY014733.1	Duck	Minnesota	1981	H1N1	Past
CY077677.1	Duck	Victoria	1981	H1N1	Past
CY004498.1	Northern Pintail	Alberta	1981	H1N5	Past
CY117091.1	Mallard	Stralsund	1981	H2N1	Past
CY117099.1	Mallard	Stralsund	1981	H2N1	Past
CY116759.1	Duck	Rugen	1981	H2N3	Past
CY117075.1	Mallard	Stralsund	1981	H2N3	Past
CY117083.1	Mallard	Stralsund	1981	H2N3	Past
CY117107.1	Duck	Rugen	1981	H2N3	Past
CY117115.1	Duck	Rugen	1981	H2N3	Past
CY117123.1	Duck	Rugen	1981	H2N3	Past
CY117131.1	Mallard	Stralsund	1981	H2N3	Past
CY117139.1	Mallard	Stralsund	1981	H2N3	Past
AF082040.1	Duck	Minnesota	1981	H5N1	Past
CY014726.1	Duck	Minnesota	1981	H5N1	Past
AB558457.1	Turkey	Minnesota	1981	H5N2	Past
CY014722.1	Duck	Minnesota	1981	H5N2	Past
CY077685.1	Duck	Victoria	1981	H5N2	Past
U79454.1	Turkey	Minnesota	1981	H5N2	Past
CY004142.1	Mallard	Alberta	1981	H6N1	Past
M35996.1	Mallard	Gurjev	1982	H14	Past
CY014604.1	Mallard	Astrakhan	1982	H14N5	Past
FJ975075.1	Herring Gull	Astrakhan	1982	H14N5	Past
CY014901.1	Mallard	New York	1982	H1N2	Past
CY014961.1	Mallard	New York	1982	H3N8	Past
CY014937.1	Northern Pintail	New York	1982	H4N3	Past
CY014929.1	Mallard	New York	1982	H4N8	Past
U69277.1	Mallard	Wisconsin	1982	H5	Past
CY014849.1	Mallard	New York	1982	H5N2	Past
CY036759.1	Mallard	Wisconsin	1982	H5N2	Past
EU743600.2	Turkey	Texas	1982	H5N2	Past
CY004178.1	Blue-winged Teal	Alberta	1982	H6N4	Past

CY004146.1	Northern Pintail	Alberta	1982	H6N6	Past
CY004154.1	Wigeon	Alberta	1982	H6N6	Past
CY004162.1	Blue-winged Teal	Alberta	1982	H6N6	Past
CY004170.1	Mallard	Alberta	1982	H6N6	Past
CY014945.1	Wood Duck	New York	1982	H6N8	Past
CY014953.1	Mallard	New York	1982	H6N8	Past
AF202236.1	Chicken	England	1982	H7N1	Past
GU186610.1	Turkey	Minnesota	1982	H7N3	Past
CY006032.1	Australian Shelduck	Western Australia	1983	H15N2	Past
AB295613.1	Duck	Australia	1983	H15N8	Past
CY006009.1	Duck	Australia	1983	H15N8	Past
L43916.1	Duck	Australia	1983	H15N8	Past
CY006033.1	Sooty Tern	Western Australia	1983	H15N9	Past
CY006034.1	Wedge-tailed Shearwater	Western Australia	1983	H15N9	Past
GU066779.1	Mallard	Marquenterre	1983	H1N1	Past
CY116859.1	Mallard	Potsdam	1983	H2N1	Past
CY005765.1	Mallard	Potsdam	1983	H2N2	Past
CY067272.1	Mallard	Postdam	1983	H2N2	Past
CY116851.1	Mallard	Potsdam	1983	H2N2	Past
CY116867.1	Mallard	Potsdam	1983	H2N2	Past
CY116875.1	Mallard	Potsdam	1983	H2N2	Past
CY116883.1	Mallard	Potsdam	1983	H2N2	Past
CY116963.1	Chicken	Jena	1983	H2N2	Past
DQ017486.1	Mallard	Postdam	1983	H2N2	Past
DQ017493.1	Mallard	Postdam	1983	H2N2	Past
L11139.1	Mallard	Potsdam	1983	H2N2	Past
CY005940.1	Blue-winged Teal	Alberta	1983	H3N1	Past
CY005941.1	Mallard	Alberta	1983	H3N5	Past
CY028267.1	Black Duck	Western Australia	1983	H3N8	Past
CY028283.1	Red-necked Stint	Western Australia	1983	H3N8	Past
GU052291.1	Duck	Washington	1983	H3N8	Past
CY005957.1	Mallard	Alberta	1983	H4N4	Past
AF082043.1	Gull	Pennsylvania	1983	H5N1	Past
EU871820.1	Turkey	Ontario	1983	H5N1	Past
AB558463.1	Chicken	Pennsylvania	1983	H5N2	Past
CY015073.1	Chicken	Pennsylvania	1983	H5N2	Past
CY107847.1	Chicken	Pennsylvania	1983	H5N2	Past
CY107848.1	Chicken	Pennsylvania	1983	H5N2	Past
GU052771.1	Chicken	Pennsylvania	1983	H5N2	Past
GU052787.1	Chicken	Pennsylvania	1983	H5N2	Past
J04325.1	Chicken	Pennsylvania	1983	H5N2	Past
M10243.1	Chicken	Pennsylvania	1983	H5N2	Past
M18001.1	Chicken	Pennsylvania	1983	H5N2	Past
CY015089.1	Turkey	Ireland	1983	H5N8	Past
GU052853.1	Duck	Ireland	1983	H5N8	Past
GU052860.1	Turkey	Ireland	1983	H5N8	Past
M18450.1	Duck	Ireland	1983	H5N8	Past

M18451.1	Turkey	Ireland	1983	H5N8	Past
CY005995.1	Mallard	Alberta	1984	H10N6	Past
CY005996.1	Northern Pintail	Alberta	1984	H10N6	Past
CY005959.1	Mallard	Alberta	1984	H4N2	Past
CY092161.1	Duck	Western Australia	1984	H4N6	Past
CY094895.1	Duck	Western Australia	1984	H4N6	Past
CY035890.1	Red-necked Stint	Western Australia	1984	H4N8	Past
GQ176120.1	Fowl	Hampshire	1985	H10N4	Past
GQ176128.1	Mallard	Gloucestershire	1985	H10N4	Past
AF091311.1	Mallard	Tennessee	1985	H1N1	Past
CY005958.1	Ruddy Turnstone	New Jersey	1985	H4N6	Past
CY005961.1	Northern Pintail	Alberta	1985	H4N6	Past
M25289.1	Ruddy Turnstone	New Jersey	1985	H4N6	Past
AY633308.1	Mallard	Alberta	1985	H6N2	Past
AY633316.1	Northern Pintail	Alberta	1985	H6N2	Past
CY004186.1	Mallard	Alberta	1985	H6N2	Past
CY004194.1	Blue-winged Teal	Alberta	1985	H6N2	Past
CY004210.1	Northern Pintail	Alberta	1985	H6N2	Past
CY004218.1	Northern Shoveler	Alberta	1985	H6N2	Past
CY005106.1	Mallard	Alberta	1985	H6N2	Past
CY004202.1	Mallard	Alberta	1985	H6N3	Past
GU050860.1	Amer Green-winged Teal	Louisiana	1987	H10N2	Past
GQ176112.1	Whistling Swan	Shimane	1988	H10N4	Past
EU743314.1	Amer Green-winged Teal	Louisiana	1988	H10N7	Past
CY089557.1	Mallard	Ohio	1989	H10N6	Past
CY017781.1	Mallard	Ohio	1989	H10N7	Past
CY020925.1	Mallard	Ohio	1989	H10N7	Past
AF091312.1	Duck	Australia	1989	H1N1	Past
CY081292.1	Mallard	Ohio	1989	mixed	Past
CY098540.1	Teal	Chany	2008	H15N4	Present
HM179251.1	Mallard	Switzerland	2009	H10	Present
CY097301.1	Mallard	Wisconsin	2009	H10N1	Present
JN817582.1	Wild Bird	Korea	2009	H10N1	Present
CY062572.1	Muscovy Duck	Thailand	2009	H10N3	Present
CY062580.1	Muscovy Duck	Thailand	2009	H10N3	Present
CY062588.1	Muscovy Duck	Thailand	2009	H10N3	Present
CY062596.1	Muscovy Duck	Thailand	2009	H10N3	Present
CY079404.1	American Coot	Mississippi	2009	H10N3	Present
CY088742.1	Duck	Thailand	2009	H10N3	Present
CY088744.1	Duck	Thailand	2009	H10N3	Present
CY088746.1	Duck	Thailand	2009	H10N3	Present
CY088748.1	Duck	Thailand	2009	H10N3	Present
CY088750.1	Duck	Thailand	2009	H10N3	Present
CY088752.1	Duck	Thailand	2009	H10N3	Present
CY088754.1	Duck	Thailand	2009	H10N3	Present
CY088756.1	Duck	Thailand	2009	H10N3	Present
CY088758.1	Duck	Thailand	2009	H10N3	Present

CY088760.1	Duck	Thailand	2009	H10N3	Present
CY088762.1	Duck	Thailand	2009	H10N3	Present
CY088764.1	Duck	Thailand	2009	H10N3	Present
CY097159.1	American Coot	Illinois	2009	H10N3	Present
CY097293.1	Lesser Scaup	Wisconsin	2009	H10N3	Present
CY097614.1	Northern Shoveler	Missouri	2009	H10N3	Present
CY078723.1	Mallard	Interior Alaska	2009	H10N5	Present
CY079396.1	Northern Shoveler	Mississippi	2009	H10N6	Present
CY093910.1	Amer Green-winged Teal	California	2009	H10N7	Present
CY097526.1	Lesser Scaup	Illinois	2009	H10N7	Present
GQ404728.1	Pekin Duck	South Africa	2009	H10N7	Present
CY094549.1	Mallard	California	2009	H1N1	Present
CY094701.1	Mallard	California	2009	H1N1	Present
CY097002.1	Mallard	Wisconsin	2009	H1N1	Present
CY097366.1	Mallard	Wisconsin	2009	H1N1	Present
CY097382.1	Mallard	Wisconsin	2009	H1N1	Present
CY097390.1	Mallard	Wisconsin	2009	H1N1	Present
CY097406.1	Mallard	Wisconsin	2009	H1N1	Present
CY097502.1	Amer Green-winged Teal	Wisconsin	2009	H1N1	Present
HM370960.1	Turkey	Ontario	2009	H1N1	Present
HM370967.1	Turkey	Ontario	2009	H1N1	Present
HM370975.1	Turkey	Ontario	2009	H1N1	Present
HM450134.1	Turkey	Ontario	2009	H1N1	Present
HQ897965.1	Mallard	Korea	2009	H1N1	Present
CY097195.1	Amer Green-winged Teal	Illinois	2009	H1N2	Present
CY097438.1	Blue-winged Teal	Wisconsin	2009	H1N2	Present
AB560965.1	Duck	Hokkaido	2009	H1N3	Present
AB560963.1	Duck	Hokkaido	2009	H1N5	Present
CY096994.1	Blue-winged Teal	Wisconsin	2009	H2N3	Present
CY097203.1	Amer Green-winged Teal	Illinois	2009	H2N3	Present
CY097334.1	Mallard	Wisconsin	2009	H2N3	Present
CY097374.1	Mallard	Wisconsin	2009	H2N3	Present
CY097598.1	Mallard	Missouri	2009	H2N3	Present
CY103519.1	Mallard	Alberta	2009	H2N3	Present
CY103527.1	Mallard	Alberta	2009	H2N3	Present
CY094637.1	Mallard	California	2009	H4N2	Present
CY094669.1	Mallard	California	2009	H4N2	Present
CY096978.1	American Black Duck	Wisconsin	2009	H4N2	Present
CY097018.1	Blue-winged Teal	Wisconsin	2009	H4N2	Present
CY097430.1	Northern Shoveler	Wisconsin	2009	H4N2	Present
CY097446.1	Mallard	Wisconsin	2009	H4N2	Present
CY097470.1	Blue-winged Teal	Wisconsin	2009	H4N2	Present
CY097478.1	Mallard	Wisconsin	2009	H4N2	Present
CY125581.1	Blue-winged Teal	New Brunswick	2009	H4N2	Present
HQ285886.1	Duck	Hunan	2009	H4N2	Present
CY078715.1	Mallard	Interior Alaska	2009	H4N6	Present
CY078731.1	Mallard	Interior Alaska	2009	H4N6	Present

CY078739.1	Mallard	Interior Alaska	2009	H4N6	Present
CY078747.1	Mallard	Interior Alaska	2009	H4N6	Present
CY078763.1	Mallard	Interior Alaska	2009	H4N6	Present
CY078771.1	Mallard	Interior Alaska	2009	H4N6	Present
CY078787.1	Mallard	Interior Alaska	2009	H4N6	Present
CY078851.1	Mallard	Interior Alaska	2009	H4N6	Present
CY079606.1	Mallard	Interior Alaska	2009	H4N6	Present
CY079792.1	Mallard	Interior Alaska	2009	H4N6	Present
CY079831.1	Mallard	Interior Alaska	2009	H4N6	Present
CY079862.1	Mallard	Interior Alaska	2009	H4N6	Present
CY079878.1	Mallard	Interior Alaska	2009	H4N6	Present
CY079886.1	Mallard	Interior Alaska	2009	H4N6	Present
CY094605.1	Mallard	California	2009	H4N6	Present
CY097010.1	Mallard	Wisconsin	2009	H4N6	Present
CY097026.1	Blue-winged Teal	Wisconsin	2009	H4N6	Present
CY097042.1	Blue-winged Teal	Wisconsin	2009	H4N6	Present
CY097285.1	Mallard	Wisconsin	2009	H4N6	Present
CY097662.1	Mallard	Ohio	2009	H4N6	Present
CY096970.1	Bufflehead	Illinois	2009	H4N8	Present
CY097542.1	Mallard	Illinois	2009	H4N8	Present
CY097550.1	Ring-necked Duck	Illinois	2009	H4N8	Present
CY097654.1	Mallard	Ohio	2009	H4N9	Present
CY125573.1	Blue-winged Teal	Prince Edward Is	2009	H4N9	Present
AB517663.1	Grey Heron	Hong Kong	2009	H5N1	Present
AB517665.1	Whooper Swan	Mongolia	2009	H5N1	Present
AB517667.1	Whooper Swan	Mongolia	2009	H5N1	Present
AB520708.1	Whooper Swan	Mongolia	2009	H5N1	Present
AB521159.1	Peregrine Falcon	Hong Kong	2009	H5N1	Present
AB521161.1	Large-billed Crow	Hong Kong	2009	H5N1	Present
AB521163.1	Crested Myna	Hong Kong	2009	H5N1	Present
AB521999.1	Bar-headed Goose	Mongolia	2009	H5N1	Present
AB522001.1	Common Goldeneye	Mongolia	2009	H5N1	Present
AB523366.1	Bar-headed Goose	Mongolia	2009	H5N1	Present
AB523368.1	Ruddy Shelduck	Mongolia	2009	H5N1	Present
AB523759.1	Ruddy Shelduck	Mongolia	2009	H5N1	Present
AB523767.1	Bar-headed Goose	Mongolia	2009	H5N1	Present
AB523775.1	Common Goldeneye	Mongolia	2009	H5N1	Present
AB530992.1	Mallard	Hokkaido	2009	H5N1	Present
AB557629.1	Feral Pigeon	Hong Kong	2009	H5N1	Present
AB557633.1	Oriental Magpie Robin	Hong Kong	2009	H5N1	Present
AB601132.1	Chicken	Egypt	2009	H5N1	Present
AB601133.1	Chicken	Egypt	2009	H5N1	Present
AB601134.1	Chicken	Egypt	2009	H5N1	Present
AB601135.1	Chicken	Egypt	2009	H5N1	Present
AB601136.1	Chicken	Egypt	2009	H5N1	Present
AB601137.1	Chicken	Egypt	2009	H5N1	Present
CY043374.1	Chicken	Bangladesh	2009	H5N1	Present

CY062601.1	Chicken	Egypt	2009	H5N1	Present
CY062602.1	Chicken	Egypt	2009	H5N1	Present
CY063318.2	Great Crested-grebe	Qinghai	2009	H5N1	Present
CY080355.1	Chicken	Sikkim	2009	H5N1	Present
CY080363.1	Chicken	West Bengal	2009	H5N1	Present
CY080371.1	Chicken	West Bengal	2009	H5N1	Present
CY095692.1	Duck	Viet Nam	2009	H5N1	Present
CY095695.1	Duck	Viet Nam	2009	H5N1	Present
CY095698.1	Duck	Viet Nam	2009	H5N1	Present
CY095701.1	Chicken	Viet Nam	2009	H5N1	Present
CY095704.1	Duck	Viet Nam	2009	H5N1	Present
CY097350.1	Mallard	Wisconsin	2009	H5N1	Present
CY098291.1	Herring Gull	Mongolia	2009	H5N1	Present
CY098293.1	Ruddy Shelduck	Mongolia	2009	H5N1	Present
FR687255.1	Chicken	Egypt	2009	H5N1	Present
GQ338084.1	Black-headed Gull	Tyva	2009	H5N1	Present
GQ338087.1	Great Crested-grebe	Tyva	2009	H5N1	Present
GQ386142.1	Grebe	Tyva	2009	H5N1	Present
GQ386150.1	Bean Goose	Tyva	2009	H5N1	Present
GQ917227.1	Chicken	India	2009	H5N1	Present
GQ917229.1	Chicken	India	2009	H5N1	Present
GQ917231.1	Chicken	India	2009	H5N1	Present
GU002672.1	Chicken	Egypt	2009	H5N1	Present
GU002673.1	Duck	Egypt	2009	H5N1	Present
GU002674.1	Duck	Egypt	2009	H5N1	Present
GU002675.1	Duck	Egypt	2009	H5N1	Present
GU002676.1	Duck	Egypt	2009	H5N1	Present
GU002677.1	Goose	Egypt	2009	H5N1	Present
GU002678.1	Duck	Egypt	2009	H5N1	Present
GU002679.1	Turkey	Egypt	2009	H5N1	Present
GU002680.1	Chicken	Egypt	2009	H5N1	Present
GU002681.1	Duck	Egypt	2009	H5N1	Present
GU002682.1	Duck	Egypt	2009	H5N1	Present
GU002683.1	Chicken	Egypt	2009	H5N1	Present
GU002684.1	Chicken	Egypt	2009	H5N1	Present
GU002685.1	Duck	Egypt	2009	H5N1	Present
GU002686.1	Duck	Egypt	2009	H5N1	Present
GU002687.1	Chicken	Egypt	2009	H5N1	Present
GU002688.1	Chicken	Egypt	2009	H5N1	Present
GU002689.1	Chicken	Egypt	2009	H5N1	Present
GU002690.1	Chicken	Egypt	2009	H5N1	Present
GU002692.1	Chicken	Egypt	2009	H5N1	Present
GU002693.1	Chicken	Egypt	2009	H5N1	Present
GU002695.1	Chicken	Egypt	2009	H5N1	Present
GU002696.1	Duck	Egypt	2009	H5N1	Present
GU002697.1	Duck	Egypt	2009	H5N1	Present
GU002698.1	Chicken	Egypt	2009	H5N1	Present

GU002699.1	Duck	Egypt	2009	H5N1	Present
GU002700.1	Chicken	Egypt	2009	H5N1	Present
GU002701.1	Turkey	Egypt	2009	H5N1	Present
GU002702.1	Turkey	Egypt	2009	H5N1	Present
GU002703.1	Chicken	Egypt	2009	H5N1	Present
GU002704.1	Duck	Egypt	2009	H5N1	Present
GU002705.1	Chicken	Egypt	2009	H5N1	Present
GU083653.1	Chicken	West Bengal	2009	H5N1	Present
GU083661.1	Chicken	West Bengal	2009	H5N1	Present
GU271998.1	Chicken	West Bengal	2009	H5N1	Present
GU272006.1	Chicken	West Bengal	2009	H5N1	Present
GU354081.1	Chicken	Sikkim	2009	H5N1	Present
GU356583.1	Chicken	West Bengal	2009	H5N1	Present
GU366078.1	Chicken	Egypt	2009	H5N1	Present
GU370127.1	Chicken	Egypt	2009	H5N1	Present
GU727677.1	Duck	Eastern China	2009	H5N1	Present
GU811717.1	Chicken	Egypt	2009	H5N1	Present
GU811718.1	Duck	Egypt	2009	H5N1	Present
GU811719.1	Chicken	Egypt	2009	H5N1	Present
GU811720.1	Chicken	Egypt	2009	H5N1	Present
GU811721.1	Chicken	Egypt	2009	H5N1	Present
GU811722.1	Chicken	Egypt	2009	H5N1	Present
GU811723.1	Duck	Egypt	2009	H5N1	Present
GU811724.1	Duck	Egypt	2009	H5N1	Present
GU811725.1	Chicken	Egypt	2009	H5N1	Present
GU811726.1	Chicken	Egypt	2009	H5N1	Present
GU811728.1	Duck	Egypt	2009	H5N1	Present
GU811729.1	Chicken	Egypt	2009	H5N1	Present
GU811731.1	Goose	Egypt	2009	H5N1	Present
GU811732.1	Duck	Egypt	2009	H5N1	Present
GU811733.1	Duck	Egypt	2009	H5N1	Present
GU811734.1	Turkey	Egypt	2009	H5N1	Present
GU811735.1	Duck	Egypt	2009	H5N1	Present
GU811737.1	Goose	Egypt	2009	H5N1	Present
GU811738.1	Duck	Egypt	2009	H5N1	Present
GU811739.1	Duck	Egypt	2009	H5N1	Present
GU811740.1	Duck	Egypt	2009	H5N1	Present
GU811741.1	Goose	Egypt	2009	H5N1	Present
GU811742.1	Chicken	Egypt	2009	H5N1	Present
GU811743.1	Goose	Egypt	2009	H5N1	Present
GU811744.1	Duck	Egypt	2009	H5N1	Present
GU811745.1	Goose	Egypt	2009	H5N1	Present
GU811746.1	Duck	Egypt	2009	H5N1	Present
GU811747.1	Chicken	Egypt	2009	H5N1	Present
HM006728.1	Bar-headed Goose	Mongolia	2009	H5N1	Present
HM006730.1	Bar-headed Goose	Mongolia	2009	H5N1	Present
HM006733.1	Common Goldeneye	Mongolia	2009	H5N1	Present

HM006735.1	Common Goldeneye	Mongolia	2009	H5N1	Present
HM006736.1	Ruddy Shelduck	Mongolia	2009	H5N1	Present
HM006740.1	Whooper Swan	Mongolia	2009	H5N1	Present
HM006741.1	Whooper Swan	Mongolia	2009	H5N1	Present
HM006742.1	Whooper Swan	Mongolia	2009	H5N1	Present
HM006743.1	Whooper Swan	Mongolia	2009	H5N1	Present
HM006745.1	Whooper Swan	Mongolia	2009	H5N1	Present
HM006746.1	Whooper Swan	Mongolia	2009	H5N1	Present
HM006747.1	Whooper Swan	Mongolia	2009	H5N1	Present
HM172074.1	Chicken	Hunan	2009	H5N1	Present
HM172081.1	Chicken	Hebei	2009	H5N1	Present
HM172093.1	Chicken	Shandong	2009	H5N1	Present
HM352796.1	Duck	Egypt	2009	H5N1	Present
HQ020367.1	Brown-headed Gull	Qinghai	2009	H5N1	Present
HQ020368.1	Brown-headed Gull	Qinghai	2009	H5N1	Present
HQ020369.1	Great Black-headed Gull	China	2009	H5N1	Present
HQ020370.1	Great Black-headed Gull	Qinghai	2009	H5N1	Present
HQ020371.1	Great Black-headed Gull	Qinghai	2009	H5N1	Present
HQ020372.1	Great Black-headed Gull	Qinghai	2009	H5N1	Present
HQ020373.1	Great Black-headed Gull	Qinghai	2009	H5N1	Present
HQ020374.1	Great Black-headed Gull	Qinghai	2009	H5N1	Present
HQ020375.1	Great Cormorant	Qinghai	2009	H5N1	Present
HQ198252.1	Chicken	Egypt	2009	H5N1	Present
HQ198253.1	Goose	Egypt	2009	H5N1	Present
HQ198254.1	Chicken	Egypt	2009	H5N1	Present
JF302895.1	Chicken	Indonesia	2009	H5N1	Present
JF357720.1	Chicken	Egypt	2009	H5N1	Present
JF357721.1	Chicken	Egypt	2009	H5N1	Present
JF357722.1	Chicken	Egypt	2009	H5N1	Present
JF357723.1	Chicken	Egypt	2009	H5N1	Present
JF510041.1	Wild Duck	Korea	2009	H5N1	Present
JF746741.1	Chicken	Egypt	2009	H5N1	Present
JF975561.1	Swan	Shanghai	2009	H5N1	Present
JN055363.1	Chicken	Vietnam	2009	H5N1	Present
JN055364.1	Muscovy Duck	Vietnam	2009	H5N1	Present
JN055365.1	Chicken	Vietnam	2009	H5N1	Present
JN055366.1	Chicken	Vietnam	2009	H5N1	Present
JN055367.1	Chicken	Vietnam	2009	H5N1	Present
JN055368.1	Muscovy Duck	Vietnam	2009	H5N1	Present
JN055369.1	Muscovy Duck	Vietnam	2009	H5N1	Present
JN055370.1	Chicken	Vietnam	2009	H5N1	Present
JN055371.1	Chicken	Vietnam	2009	H5N1	Present
JN055372.1	Chicken	Vietnam	2009	H5N1	Present
JN055373.1	Chicken	Vietnam	2009	H5N1	Present
JN055374.1	Chicken	Vietnam	2009	H5N1	Present
JN055375.1	Chicken	Vietnam	2009	H5N1	Present
JN055376.1	Chicken	Vietnam	2009	H5N1	Present

JN055377.1	Chicken	Vietnam	2009	H5N1	Present
JN055378.1	Chicken	Vietnam	2009	H5N1	Present
JN055379.1	Chicken	Vietnam	2009	H5N1	Present
JN055380.1	Chicken	Vietnam	2009	H5N1	Present
JN055381.1	Chicken	Vietnam	2009	H5N1	Present
JN055382.1	Chicken	Vietnam	2009	H5N1	Present
JN055383.1	Chicken	Vietnam	2009	H5N1	Present
JN055384.1	Chicken	Vietnam	2009	H5N1	Present
JN055385.1	Duck	Vietnam	2009	H5N1	Present
JN055386.1	Duck	Vietnam	2009	H5N1	Present
JN055387.1	Duck	Vietnam	2009	H5N1	Present
JN055388.1	Duck	Vietnam	2009	H5N1	Present
JN055389.1	Duck	Vietnam	2009	H5N1	Present
JN055390.1	Duck	Vietnam	2009	H5N1	Present
JN055391.1	Duck	Vietnam	2009	H5N1	Present
JN588811.1	Chicken	Cambodia	2009	H5N1	Present
JN588812.1	Chicken	Cambodia	2009	H5N1	Present
JN714467.1	Chicken	Egypt	2009	H5N1	Present
JX021303.1	Duck	Vietnam	2009	H5N1	Present
CY094013.1	Mallard	California	2009	H5N2	Present
CY094581.1	Mallard	California	2009	H5N2	Present
CY094589.1	Mallard	California	2009	H5N2	Present
CY094597.1	Mallard	California	2009	H5N2	Present
CY094613.1	Mallard	California	2009	H5N2	Present
CY094629.1	Mallard	California	2009	H5N2	Present
CY094645.1	Mallard	California	2009	H5N2	Present
CY094653.1	Mallard	California	2009	H5N2	Present
CY094661.1	Mallard	California	2009	H5N2	Present
CY094693.1	Mallard	California	2009	H5N2	Present
CY094709.1	Mallard	California	2009	H5N2	Present
CY094717.1	Mallard	California	2009	H5N2	Present
CY094725.1	Mallard	California	2009	H5N2	Present
CY094733.1	Mallard	California	2009	H5N2	Present
CY097176.1	Mallard	Illinois	2009	H5N2	Present
CY120707.1	Mallard	California	2009	H5N2	Present
GU086232.1	Wild Bird	Korea	2009	H5N2	Present
JQ737229.1	Mallard	Czech Republic	2009	H5N3	Present
GU727661.1	Duck	Eastern China	2009	H5N5	Present
CY094573.1	Amer Green-winged Teal	California	2009	H7N3	Present
CY097622.1	Mallard	Missouri	2009	H7N3	Present
AB538456.1	Quail	Aichi	2009	H7N6	Present
AB538457.1	Quail	Aichi	2009	H7N6	Present
AB538458.1	Quail	Aichi	2009	H7N6	Present
AB538459.1	Quail	Aichi	2009	H7N6	Present
AB538460.1	Quail	Aichi	2009	H7N6	Present
AB538461.1	Quail	Aichi	2009	H7N6	Present
AB558265.1	Duck	Chiba	2009	H7N7	Present

CY079308.1	Amer Green-winged Teal	Mississippi	2009	H7N7	Present
CY079412.1	Northern Shoveler	Mississippi	2009	H7N7	Present
HQ283357.1	Swan	Slovenia	2009	H7N7	Present
JN244227.1	Wild Bird	Korea	2009	H7N7	Present
JN244228.1	Wild Bird	Korea	2009	H7N7	Present
JN244229.1	Northern Pintail	Korea	2009	H7N7	Present
GU060482.1	Goose	Czech Republic	2009	H7N9	Present
HQ244415.1	Goose	Czech Republic	2009	H7N9	Present
CY097398.1	Mallard	Wisconsin	2009	mixed	Present
CY103500.1	Shorebird	Delaware Bay	2009	mixed	Present
JN817574.1	Wild Bird	Korea	2010	H10N1	Present
JN817575.1	Wild Bird	Korea	2010	H10N1	Present
JN817576.1	Mallard	Korea	2010	H10N6	Present
CY094765.1	Mallard	California	2010	H10N7	Present
CY094789.1	Mallard	California	2010	H10N7	Present
CY097143.1	Northern Shoveler	Mississippi	2010	H10N7	Present
CY097670.1	Northern Shoveler	Mississippi	2010	H10N7	Present
CY097694.1	Mallard	Mississippi	2010	H10N7	Present
CY120547.1	Mallard	California	2010	H10N7	Present
CY120691.1	Mallard	California	2010	H10N7	Present
CY120699.1	Mallard	California	2010	H10N7	Present
JN817572.1	Mallard	Korea	2010	H10N8	Present
CY097762.1	Mallard	Mississippi	2010	H1N1	Present
CY097770.1	Mallard	Mississippi	2010	H1N1	Present
CY120611.1	Mallard	California	2010	H1N1	Present
CY120651.1	Northern Pintail	California	2010	H1N1	Present
CY097734.1	Northern Shoveler	Mississippi	2010	H1N3	Present
CY120659.1	Northern Pintail	California	2010	H1N3	Present
HQ336713.1	Duck	Nanjing	2010	H1N3	Present
HQ336721.1	Duck	Nanjing	2010	H1N3	Present
CY120563.1	Mallard	California	2010	H2N3	Present
CY121983.1	Northern Shoveler	Georgia	2010	H2N3	Present
CY122292.1	Mallard	Netherlands	2010	H2N3	Present
CY122308.1	Mallard	Netherlands	2010	H2N3	Present
HQ165996.1	Chicken	Pakistan	2010	H3N1	Present
JN244246.1	Duck	Korea	2010	H3N6	Present
CY096656.1	Blue-winged Teal	Guatemala	2010	H3N8	Present
CY097678.1	Amer Green-winged Teal	Mississippi	2010	H3N8	Present
CY097702.1	Mallard	Mississippi	2010	H3N8	Present
CY097710.1	Mallard	Mississippi	2010	H3N8	Present
HQ874606.1	Chicken	Guangxi	2010	H3N8	Present
JF789610.1	Mallard	Czech Republic	2010	H3N8	Present
CY120603.1	Northern Shoveler	California	2010	H4N3	Present
CY094749.1	Mallard	California	2010	H4N6	Present
CY094757.1	Mallard	California	2010	H4N6	Present
CY094773.1	Mallard	California	2010	H4N6	Present
CY094781.1	Mallard	California	2010	H4N6	Present

CY120579.1	Mallard	California	2010	H4N6	Present
CY120683.1	Mallard	California	2010	H4N6	Present
CY120595.1	Mallard	California	2010	H6N1	Present
JQ990779.1	Turkey	France	2010	H6N1	Present
CY096053.1	Rosy-billed Pochard	Argentina	2010	H6N2	Present
CY120571.1	Mallard	California	2010	H6N4	Present
CY110941.1	Duck	Hubei	2010	H6N6	Present
CY110949.1	Duck	Hubei	2010	H6N6	Present
CY110973.1	Duck	Hubei	2010	H6N6	Present
JF965138.1	Goose	Eastern China	2010	H6N6	Present
JF965143.1	Chicken	Eastern China	2010	H6N6	Present
JF965155.1	Duck	Eastern China	2010	H6N6	Present
JF965156.1	Duck	Eastern China	2010	H6N6	Present
JF965157.1	Duck	Eastern China	2010	H6N6	Present
JF965167.1	Duck	Eastern China	2010	H6N6	Present
CY120643.1	Mallard	California	2010	H6N8	Present
JQ737237.1	Mallard	Czech Republic	2010	H6N9	Present
JQ973643.1	Baer's Pochard	HuNan	2010	H7N1	Present
JX307167.1	Duck	Thailand	2010	H7N4	Present
JX307213.1	Duck	Thailand	2010	H7N4	Present
CY120555.1	Mallard	California	2010	H7N5	Present
JN244238.1	Duck	Korea	2010	H7N6	Present
JX307118.1	Duck	Thailand	2010	H7N6	Present
JX307120.1	Duck	Thailand	2010	H7N6	Present
JX307130.1	Duck	Thailand	2010	H7N6	Present
JX307147.1	Duck	Thailand	2010	H7N6	Present
JX307150.1	Duck	Thailand	2010	H7N6	Present
JX307152.1	Duck	Thailand	2010	H7N6	Present
JX307159.1	Duck	Thailand	2010	H7N6	Present
JX307175.1	Duck	Thailand	2010	H7N6	Present
JX307178.1	Duck	Thailand	2010	H7N6	Present
JX307182.1	Duck	Thailand	2010	H7N6	Present
JX307189.1	Duck	Thailand	2010	H7N6	Present
JX307192.1	Duck	Thailand	2010	H7N6	Present
JX307201.1	Duck	Thailand	2010	H7N6	Present
JX307216.1	Duck	Thailand	2010	H7N6	Present
JX307223.1	Duck	Thailand	2010	H7N6	Present
JX307226.1	Duck	Thailand	2010	H7N6	Present
JX307228.1	Duck	Thailand	2010	H7N6	Present
JX307245.1	Duck	Thailand	2010	H7N6	Present
JX307255.1	Duck	Thailand	2010	H7N6	Present
AB622425.1	Duck	Hokkaido	2010	H7N7	Present
JN244230.1	Mallard	Korea	2010	H7N7	Present
JN244239.1	Duck	Korea	2010	H7N7	Present
JN244240.1	Duck	Korea	2010	H7N7	Present
JN244241.1	Duck	Korea	2010	H7N7	Present
JN244243.1	Duck	Korea	2010	H7N7	Present

JN244245.1	Duck	Korea	2010	H7N7	Present
JN244247.1	Wild Bird	Korea	2010	H7N7	Present
CY094741.1	Mallard	California	2010	mixed	Present
