# Supplementary material for: Evidence for the Circulation and Inter-Hemispheric Movement of the H14 Subtype Influenza A Virus
Source: PLoS One. 2013 Mar 28;8(3):e59216. doi: 10.1371/journal.pone.0059216 (PMC3610705; doi:10.1371/journal.pone.0059216)
Supplement: Table S2 — List of nonstructural segment sequences. List of the NS segments used in the phylogeny constructed in this study. Sequences were selected based on a BLAST analysis of the A/10OS4225/LTDU/2010 NS segment and subsequent identification of the closest 250 sequences available on GenBank. Accession numbers highlighted (Gray) represent sequences that were included in the rare lineage clade with the A/10OS4225/LTDU/2010 NS segment. (PDF) [file pone.0059216.s002.pdf]

Accession_Number	Common_Name	Location	Year	Subtype
AB259716.1	Duck	Hokkaido	2004	H5N1
AB263196.1	Duck	Mongolia	2001	H5N1
AB268556.2	Duck	Mongolia	2001	H7N1
AB304151.1	Duck	Shiga	2004	H4N6
AB473547.1	Duck	Mongolia	2002	H7N1
AB569483.1	Duck	Zambia	2008	H6N2
AB569491.1	Duck	Zambia	2008	H6N2
AB569507.1	Goose	Zambia	2008	H3N8
AB569515.1	Goose	Zambia	2008	H3N8
AB569531.1	Duck	Zambia	2009	H6N2
AB569539.1	Goose	Zambia	2009	H11N9
AB569547.1	Duck	Zambia	2009	H6N2
AB569555.1	Duck	Zambia	2009	H11N9
AB569563.1	Duck	Zambia	2009	H11N9
AB569571.1	Pelican	Zambia	2009	H9N1
AF262212.1	Chicken	Taiwan	1999	H6N1
AF523517.1	Duck	Hong Kong	1979	H9N2
AJ410596.1	Duck	Hong Kong	1977	H6N1
AJ410597.1	Goose	Hong Kong	1977	H6N4
AJ410598.1	Chicken	Hong Kong	1977	H6N4
AJ619678.1	Chicken	Germany	2003	H7N7
AM913986.1	Teal	Germany	2005	H5N1
AY342422.1	Human	Netherlands	2003	H7N7
AY342423.1	Human	Netherlands	2003	H7N7
AY342424.1	Chicken	Netherlands	2003	H7N7
AY586442.1	Mallard	Italy	2001	H7N3
AY586443.1	Mallard	Italy	2001	H7N3
AY586444.1	Turkey	Italy	2002	H7N3
AY586446.1	Turkey	Italy	2002	H7N3
AY856866.1	Duck	Shandong	2004	H5N1
AY950259.1	Chicken	Henan	2004	H5N1
AY950263.1	Wild Duck	Guangdong	2004	H5N1
CY005393.1	Mallard	Astrakhan	1982	H14N5
CY005396.1	Mallard	Astrakhan	1982	H14N6
CY005399.1	Mallard	Astrakhan	1982	H14N5
CY005434.1	Duck	Nanchang	2000	H2N9
CY005441.1	Duck	Nanchang	2000	H2N9
CY005450.1	Chicken	Nanchang	2000	H3N6
CY005457.1	Quail	Nanchang	2000	H3N6
CY005466.1	Duck	Nanchang	2000	H3N6
CY005476.1	Duck	Nanchang	1993	H4N4
CY005479.1	Duck	Nanchang	1997	H4N6
CY005482.1	Chicken	Nanchang	2000	H4N6
CY005485.1	Quail	Nanchang	2000	H4N6
CY005490.1	Duck	Nanchang	2000	H4N6
CY005497.1	Duck	Nanchang	1992	H7N1

CY005534.1	Duck	Nanchang	1992	H11N2
CY005542.1	Duck	Hong Kong	1978	H2N2
CY005550.1	Duck	Hong Kong	1978	H2N9
CY005561.1	Goose	Hong Kong	1976	H3N2
CY005571.1	Duck	Hong Kong	1978	H4N6
CY005586.1	Goose	Hong Kong	1978	H5N3
CY005601.1	Duck	Hong Kong	1976	H6N1
CY005609.1	Chicken	Hong Kong	1977	H6N1
CY005628.1	Duck	Hong Kong	1976	H4N2
CY005635.1	Duck	Hong Kong	1979	H9N2
CY005643.1	Duck	Hong Kong	1977	H9N6
CY005649.1	Duck	Hong Kong	1979	H10N9
CY005768.1	Mallard	Potsdam	1983	H2N2
CY005816.1	Duck	Ukraine	1963	H3N8
CY005855.1	Northern Shoveler	Netherlands	1999	H11N9
CY006039.1	Duck	Czechoslovakia	1956	H4N6
CY014683.1	Duck	England	1956	H11N6
CY014713.1	Duck	Germany	1973	H2N3
CY015069.1	Turkey	England	1963	H7N3
CY015085.1	Chicken	Scotland	1959	H5N1
CY015093.1	Turkey	Ireland	1983	H5N8
CY015119.1	Chicken	Italy	1997	H5N2
CY015131.1	Ruddy Turnstone	Delaware	1998	H6N8
CY022625.1	Chicken	Italy	1998	H5N9
CY031263.1	Duck	Hong Kong	1979	H9N2
CY031271.1	Duck	Hong Kong	1979	H9N2
CY031279.1	Duck	Hong Kong	1979	H9N2
CY031287.1	Duck	Hong Kong	1979	H9N2
CY041238.1	Mallard	Netherlands	2007	H2N2
CY041254.1	Mallard	Netherlands	2005	H4N2
CY041262.1	Common Teal	Netherlands	2005	H8N4
CY041278.1	Bewick's swan	Netherlands	2007	H9N2
CY041334.1	Mallard	Sweden	2005	H2N3
CY041342.1	Common Eider	Netherlands	2006	H3N8
CY043828.1	Mallard	Sweden	2005	H6N1
CY043852.1	Mallard	Netherlands	2006	H8N4
CY043876.2	Mallard	Sweden	2005	H10N4
CY055179.1	Aquatic Bird	India	2007	H11N1
CY060202.1	Greylag Goose	Netherlands	1999	H6N1
CY060209.1	Mallard	Netherlands	1999	H1N8
CY060216.1	Mallard	Sweden	2002	H11N7
CY060230.1	Mallard	Netherlands	1999	H11N9
CY060298.1	Mallard	Sweden	2002	H11N9
CY060304.1	Mallard	Sweden	2002	H10N2
CY060312.1	Mallard	Sweden	2002	H3N8
CY060320.1	Mallard	Sweden	2003	H6N2
CY060327.1	Mallard	Sweden	2002	H11N2

CY060339.1	Mallard	Sweden	2003	H11N1
CY060346.1	Mallard	Netherlands	2001	H3N6
CY060362.1	Mallard	Sweden	2002	H10N9
CY060370.1	Mallard	Sweden	2002	H2N3
CY060378.1	Mallard	Sweden	2003	H10N8
CY060407.1	Mallard	Sweden	2003	H8N4
CY060413.1	Mallard	Sweden	2002	H1N6
CY060420.1	Northern Shoveler	Netherlands	1999	H11N9
CY060435.1	White-fronted Goose	Netherlands	1999	H6N1
CY060443.1	White-fronted Goose	Netherlands	1999	H6N2
CY061889.1	Teal	Germany	2005	H5N1
CY073456.1	Swine	South Korea	2001	H11N6
CY073804.1	Duck	Malaysia	2001	H9N2
CY076896.1	Duck	Guangxi	2008	H4N2
CY076901.1	Mallard	Netherlands	2006	H1N4
CY076909.1	Mallard	Netherlands	2006	H3N1
CY076925.1	Mallard	Netherlands	2007	H4N2
CY076972.1	Mallard	Netherlands	2005	H12N8
CY077044.1	Mute Swan	Netherlands	2006	H10N7
CY077060.1	Mallard	Netherlands	2006	H11N9
CY077509.1	Mallard	New Zealand	2004	H4N6
CY079215.1	Avian	Japan	2008	H6N8
CY079223.1	Avian	Japan	2008	H4N6
CY079247.1	Avian	Japan	2008	H6N5
CY079271.1	Avian	Japan	2008	H3N6
CY081279.1	Duck	Potsdam	1980	H7N7
CY083000.1	Swine	South Korea	2001	H11N6
CY083001.1	Swine	South Korea	2001	H11N6
CY083004.1	Swine	South Korea	2001	H11N6
CY088725.1	Avian	Japan	2008	H4N2
CY092173.1	Duck	Western Australia	1984	H4N6
CY098226.1	Aquatic Bird	Korea	2009	H6N2
CY098528.1	Teal	Chany	2009	H8N8
CY098536.1	Aquatic Bird	Korea	2005	H6N5
CY100636.1	Mallard	SanJiang	2006	H3N8
CY110937.1	Duck	Taiwan	2005	H5N2
CY116727.1	Peking duck	Wagun	1984	H11N2
CY116737.1	Mallard	Berlin	1980	mixed
CY116746.1	Mallard	Rugen	1980	mixed
CY116755.1	Mallard	Stralsund	1981	H1
CY116855.1	Mallard	Potsdam	1983	H2N2
CY116863.1	Mallard	Potsdam	1983	H2N1
CY116895.1	Duck	Hong Kong	1978	H2N2
CY116959.1	Chicken	Jena	1984	H2N2
CY117071.1	Mallard	Rugen	1980	H2N3
CY117095.1	Mallard	Stralsund	1981	H2N1
CY117103.1	Mallard	Stralsund	1981	H2N1

CY117151.1	Peking duck	Wagun	1985	H2N3
CY117175.1	Peking duck	Heinersdorf	1985	H2N3
CY121963.1	White-fronted Goose	Netherlands	1999	H2N2
CY121987.1	Northern Shoveler	Georgia	2010	H2N3
CY121995.1	Mallard	Netherlands	2001	H2N3
CY122224.1	Mallard	Sweden	2005	H2N9
CY122272.1	Mallard	Netherlands	2008	H2N3
CY125011.1	Duck	Jiangsu	2011	H11N3
DQ017490.1	Mallard	Postdam	1983	H2N2
DQ017498.1	Mallard	Postdam	1983	H2N2
DQ251446.1	Mallard	Denmark	2003	H5N7
DQ376765.1	Chicken	Taiwan	1997	H6N1
DQ376766.1	Chicken	Taiwan	1998	H6N1
DQ376767.1	Chicken	Taiwan	1999	H6N1
DQ376768.1	Chicken	Taiwan	1999	H6N1
DQ376769.1	Partridge	Taiwan	1999	H6N1
DQ376770.1	Chicken	Taiwan	1999	H6N1
DQ376771.1	Duck	Taiwan	1999	H6N1
DQ376772.1	Chicken	Taiwan	2000	H6N1
DQ376773.1	Duck	Taiwan	2000	H6N1
DQ376774.1	Chicken	Taiwan	2001	H6N1
DQ376776.1	Chicken	Taiwan	2001	H6N1
DQ376779.1	Chicken	Taiwan	2002	H6N1
DQ376781.1	Chicken	Taiwan	2002	H6N1
DQ376786.1	Chicken	Taiwan	2002	H6N1
DQ376790.1	Duck	Taiwan	2003	H6N1
DQ376795.1	Duck	Taiwan	2004	H6N5
DQ486128.1	Mallard	Xuyi	2004	H11N?
DQ997114.1	Chicken	Hubei	1997	H5N1
DQ997117.1	Chicken	Hubei	1997	H5N1
DQ997127.1	Chicken	Hubei	1997	H5N1
DQ997136.1	Chicken	Hubei	1997	H5N1
DQ997213.1	Mallard	Guangxi	2004	H5N1
DQ997222.1	Chicken	Henan	2004	H5N1
DQ997311.1	Chicken	Jilin	2002	H5N1
DQ997484.1	Chicken	Hebei	2002	H9N2
EF597395.1	Migratory Duck	Hong Kong	2005	H5N8
EU152241.1	Gull	Moscow	2006	H6N2
EU158148.1	Turkey	Italy	2003	H7N3
EU249549.1	Duck	Malaysia	2004	H5N2
EU263357.1	Duck	Guangxi	2003	H5N1
EU518725.1	Mallard	Sweden	2005	H3N8
EU518731.1	Mallard	Sweden	2005	H3N8
EU518752.1	Mallard	Sweden	2005	H2N3
EU580556.1	Garganey	Astrakhan	2002	H4N8
EU880346.1	Mallard	Yan chen	2005	H4N6
FJ349250.1	Mallard	Zha C43Long	2004	H4N6

FJ432766.1	Duck	Italy	2006	H3N2
FJ432782.1	Goose	Italy	2003	H1N1
FJ434368.2	Garganey	Korgalzhyn	2004	H3N6
FJ750858.1	Magpie	Korea	2007	H7N7
FJ750868.1	Mallard	Korea	2007	H7N7
FJ959090.1	Mallard	Korea	2007	H7N7
GQ162789.1	Duck	Primorie	2001	H5N2
GQ176116.1	Fowl	Hampshire	1985	H10N4
GQ219714.1	Spotbill Duck	Xuyi	2005	H11N2
GQ219715.1	Baikal Teal	Hongze	2005	H11N9
GQ227609.1	Duck	Primorie	2001	H5N3
GQ290471.1	Environment	Hunan	2007	H10N8
GQ325641.1	Environment	Hunan	2007	H10N8
GQ325649.1	Environment	Hunan	2007	H10N8
GQ325657.1	Environment	Hunan	2007	H10N8
GQ907290.1	Bar-headed Goose	Mongolia	2005	H12N3
GQ907330.1	Red-creasted Pochard	Mongolia	2006	H3N6
GQ907338.1	Ruddy Shelduck	Mongolia	2005	H12N3
GQ907346.1	Ruddy Shelduck	Mongolia	2005	H12N3
GQ907354.1	Whooper Swan	Mongolia	2005	H12N3
GU050306.1	Avian	Egypt	2006	H9N2
GU052212.1	Northern Shoveler	Netherlands	1999	H11N7
GU052256.1	Mallard	Gurjev	1982	H14N5
GU052584.1	Mallard	Netherlands	2005	H5N2
GU052811.1	Whistling Swan	Shimone	1983	H5N3
GU052872.1	Mallard	Sweden	2002	H5N9
GU053125.1	Human	Netherlands	2003	H7N7
GU066782.1	Mallard	Marquenterre	1983	H1N1
GU182186.1	environment	Hunan	2008	H5N1
GU215038.1	Swine	Jilin	2008	H3N2
HM144901.1	Mallard	Jiangxi	2005	H6N1
HM745402.1	Duck	Jiangxi	2009	H11N2
HM849007.1	Tufted Duck	Portugal	2006	H7N3
HM849021.1	Mallard	Portugal	2006	H4N6
HQ166000.1	Chicken	Pakistan	2010	H3N1
HQ259228.1	Mallard	Bavaria	2008	H1N1
HQ259236.1	Mallard	Bavaria	2008	H1N1
HQ285890.1	Duck	Hunan	2009	H4N2
HQ897969.1	Mallard	Korea	2009	H1N1
JN029553.1	Whooper Swan	Mongolia	2007	H3N8
JN029606.1	environment	Mongolia	2007	H4N6
JN029612.1	Northern Pintail	Mongolia	2007	H4N2
JN029627.1	Northern Pintail	Mongolia	2007	H3N8
JN029638.1	Canvasback	Mongolia	2007	H10N6
JN029646.1	Ruddy Shelduck	Mongolia	2007	H3N8
JN029689.1	Wild Duck	Mongolia	2008	H7N9
JN087039.1	environment	Korea	2003	H3N2

JN087076.1	environment	Korea	2004	H3N3
JN087092.1	Duck	Korea	2004	H3N2
JN087100.1	Duck	Korea	2004	H3N2
JN966909.1	Swan	Czech Republic	2011	H7N7
JQ364985.1	Chicken	Iran	1998	H9N2
JQ924793.1	Duck	Guangdong	2012	H10N8
JX030402.1	Mallard	Korea	2010	H13N2
JX151011.1	Swine	Guangdong	2011	H4N8
JX308782.1	Chicken	Iran	1998	H9N2
JX500447.1	Swine	Hubei	2008	H10N5
M60800.1	Northern Pintail	Primorje	1976	H2N3
M80942.1	Budgerigar	Hokkaido	1977	H4N6
M80966.1	Swine	Netherlands	1980	H1N1
M80968.1	Swine	China	1978	H3N2
U49493.1	Duck	Nanchang	1993	H7N4
Z26864.1	oystercatcher	Germany	1987	H1N1
