# Supplementary material for: Evidence for the Circulation and Inter-Hemispheric Movement of the H14 Subtype Influenza A Virus
Source: PLoS One. 2013 Mar 28;8(3):e59216. doi: 10.1371/journal.pone.0059216 (PMC3610705; doi:10.1371/journal.pone.0059216)
Supplement: Table S3 — List of neuraminidase segment sequences. List of the NA subtype 6 segments used in the phylogeny constructed in this study. Sequences were selected based on a BLAST analysis of the A/10OS3912/LTDU/2010 and A/10OS4225/LTDU/2010 NA segments and subsequent identification of the closest 250 sequences available on GenBank. (PDF) [file pone.0059216.s003.pdf]

Accession_Number	Common_Name	Location	Year	Subtype
FJ434372.1	Common Pochard	Aktau	2006	H4N6
FJ434371.1	Coot	Aktau	2006	H4N6
AY207535.1	Murre	Alaska	1976	H1N6
CY049782.1	Garganey	Altai	2007	H3N6
CY049766.1	Mallard	Altai	2007	H3N6
EU580571.1	Muskrat	Buryatiya	2000	H4N6
GU052361.1	Muskrat	Buryatiya	2000	H4N6
EU580572.1	Pochard	Buryatiya	2000	H4N6
GU052353.1	Pochard	Buryatiya	2000	H4N6
GU052376.1	Pochard	Buryatiya	2000	H4N6
EU580574.1	Shoveler	Buryatiya	2000	H4N6
GU052369.1	Shoveler	Buryatiya	2001	H4N6
EU580575.1	Tufted Duck	Buryatiya	2000	H4N6
EU580573.1	Pochard	Burytiya	2000	H4N6
AB295612.1	Duck	Czech	1956	H4N6
CY045329.1	Duck	Czech Republic	1956	H4N6
HQ244425.1	Mallard	Czech Republic	2007	H4N6
JF789620.1	Mallard	Czech Republic	2010	H4N6
GU052383.1	Duck	Czechoslovakia	1956	H4N6
CY101092.1	Ruddy Turnstone	Delaware	1990	mixed
EU429706.1	Duck	Eastern China	2002	H4N6
EU429713.1	Duck	Eastern China	2003	H4N6
EU429746.1	Duck	Eastern China	2004	H4N6
EU429789.1	Duck	Eastern China	2007	H4N6
EU429790.1	Duck	Eastern China	2007	H4N6
EU429792.1	Duck	Eastern China	2004	H4N6
CY014681.1	Duck	England	1956	H11N6
EU429795.1	Duck	England	1956	H11N6
CY109500.1	Duck	Fujian	2006	H6N6
CY109516.1	Duck	Fujian	2006	H6N6
CY109524.1	Duck	Fujian	2006	H6N6
CY109532.1	Duck	Fujian	2006	H6N6
CY109548.1	Duck	Fujian	2006	H6N6
CY109556.1	Duck	Fujian	2006	H6N6
CY109572.1	Duck	Fujian	2006	H6N6
CY109580.1	Duck	Fujian	2006	H6N6
CY109596.1	Duck	Fujian	2006	H6N6
CY109884.1	Duck	Fujian	2007	H6N6
CY110054.1	Duck	Fujian	2006	H6N6
CY110056.1	Duck	Fujian	2006	H6N6
CY110058.1	Duck	Fujian	2006	H6N6
CY110060.1	Duck	Fujian	2006	H6N6
CY110062.1	Duck	Fujian	2006	H6N6
CY110064.1	Duck	Fujian	2006	H6N6
CY110066.1	Duck	Fujian	2006	H6N6
CY110070.1	Duck	Fujian	2006	H6N6

CY110074.1	Duck	Fujian	2006	H6N6
CY110076.1	Duck	Fujian	2006	H6N6
CY110078.1	Duck	Fujian	2006	H6N6
CY110086.1	Duck	Fujian	2006	H6N6
CY110088.1	Duck	Fujian	2006	H6N6
CY110108.1	Duck	Fujian	2007	H6N6
CY110110.1	Duck	Fujian	2007	H6N6
CY110132.1	Duck	Fujian	2007	H6N6
CY110148.1	Duck	Fujian	2007	H6N6
CY110150.1	Duck	Fujian	2007	H6N6
CY110152.1	Duck	Fujian	2007	H6N6
CY110510.1	Duck	Fujian	2007	H6N6
CY110592.1	Duck	Fujian	2006	H6N6
CY110600.1	Duck	Fujian	2006	H6N6
CY110608.1	Duck	Fujian	2006	H6N6
CY110616.1	Duck	Fujian	2006	H6N6
CY110624.1	Duck	Fujian	2006	H6N6
CY110632.1	Duck	Fujian	2006	H6N6
CY110640.1	Duck	Fujian	2006	H6N6
CY110648.1	Duck	Fujian	2006	H6N6
CY110672.1	Duck	Fujian	2007	H6N6
CY110676.1	Duck	Fujian	2007	H6N6
CY110700.1	Duck	Fujian	2007	H6N6
CY110724.1	Duck	Fujian	2007	H6N6
HM144718.1	Duck	Fujian	2005	H6N6
GU595163.1	Muscovy Duck	Fujian	2008	H6N6
AM933235.1	Mallard	Germany	2003	H4N6
CY109924.1	Duck	Guizhou	2007	H6N6
CY110476.1	Duck	Guizhou	2007	H6N6
CY005564.1	Duck	Hong Kong	1977	H3N6
CY005641.1	Duck	Hong Kong	1977	H9N6
AY207548.1	Budgergiar	Hokkaido	1977	H4N6
AB450440.1	Duck	Hokkaido	2007	H4N6
AB292661.1	Duck	Hong Kong	1976	H3N6
CY005569.1	Duck	Hong Kong	1978	H4N6
CY109930.1	Duck	Hunan	2007	H6N6
AB472029.1	Duck	Ibaraki	2005	H4N6
JX310063.1	Chicken	India	2009	H4N6
JX310064.1	Chicken	India	2009	H4N6
JX310060.1	Duck	India	2009	H4N6
CY079221.1	Avian	Japan	2008	H4N6
CY079269.1	Avian	Japan	2008	H3N6
CY088715.1	Avian	Japan	2008	H4N6
HM144710.1	Mallard	Jiangxi	2004	H6N6
JN087218.1	Domestic Mallard	Korea	2007	H3N6
JN087114.1	Duck	Korea	2007	H3N6
JN087122.1	Duck	Korea	2007	H3N6

JN087162.1	Duck	Korea	2007	H3N6
JN087194.1	Duck	Korea	2007	H3N6
JN087202.1	Duck	Korea	2007	H3N6
JN087210.1	Duck	Korea	2007	H3N6
JN087226.1	Duck	Korea	2007	H3N6
JN244207.1	Duck	Korea	2010	H7N6
JN244208.1	Duck	Korea	2010	H3N6
JN087014.1	Environment	Korea	2002	H3N6
JN087082.1	Environment	Korea	2004	H3N6
JN817554.1	Mallard	Korea	2010	H10N6
JN817553.1	Wild Bird	Korea	2009	H10N6
CY073454.1	Swine	Korea	2001	H11N6
CY014696.1	Gull	Maryland	1977	H13N6
CY089639.1	Gull	Maryland	1977	H13N6
AB450449.1	Duck	Mongolia	2007	H7N6
AB701295.1	Duck	Mongolia	2011	H4N6
GQ907328.1	Red-crested Pochard	Mongolia	2006	H3N6
CY005449.1	Chicken	Nanchang	2000	H3N6
CY005464.1	Duck	Nanchang	2000	H3N6
CY005488.1	Duck	Nanchang	2000	H4N6
CY005461.1	Pigeon	Nanchang	2000	H3N6
AY207556.1	Quail	Nanchang	2000	H4N6
CY005455.1	Quail	Nanchang	2000	H3N6
CY005484.1	Quail	Nanchang	2000	H4N6
CY060236.1	Mallard	Netherlands	2002	H4N6
CY060243.1	Mallard	Netherlands	1999	H4N6
CY076954.1	Mallard	Netherlands	2006	H10N6
CY101621.1	Herring Gull	New Jersey	1990	H3N6
CY101108.1	Ruddy Turnstone	New Jersey	1990	H3N6
CY101120.1	Semi-palmated Sandpiper	New Jersey	1990	H3N6
CY101637.1	Semi-palmated Sandpiper	New Jersey	1990	H3N6
FM179763.1	Mallard	Norway	2006	H4N6
CY005771.1	Duck	Potsdam	1984	H5N6
HM849019.1	Mallard	Portugal	2006	H4N6
CY109364.1	Duck	Shantou	2006	H6N6
CY109404.1	Duck	Shantou	2006	H6N6
CY109452.1	Duck	Shantou	2006	H6N6
CY109460.1	Duck	Shantou	2006	H6N6
CY109732.1	Duck	Shantou	2007	H6N6
CY109740.1	Duck	Shantou	2007	H6N6
CY109756.1	Duck	Shantou	2007	H6N6
CY109772.1	Duck	Shantou	2007	H6N6
CY109946.1	Duck	Shantou	2006	H6N6
CY109954.1	Duck	Shantou	2006	H6N6
CY109956.1	Duck	Shantou	2006	H6N6
CY109958.1	Duck	Shantou	2006	H6N6
CY109964.1	Duck	Shantou	2006	H6N6

CY109974.1	Duck	Shantou	2006	H6N6
CY109976.1	Duck	Shantou	2006	H6N6
CY109982.1	Duck	Shantou	2006	H6N6
CY109984.1	Duck	Shantou	2006	H6N6
CY109986.1	Duck	Shantou	2006	H6N6
CY109996.1	Duck	Shantou	2006	H6N6
CY109998.1	Duck	Shantou	2006	H6N6
CY110000.1	Duck	Shantou	2006	H6N6
CY110002.1	Duck	Shantou	2006	H6N6
CY110008.1	Duck	Shantou	2006	H6N6
CY110012.1	Duck	Shantou	2006	H6N6
CY110014.1	Duck	Shantou	2006	H6N6
CY110016.1	Duck	Shantou	2006	H6N6
CY110018.1	Duck	Shantou	2006	H6N6
CY110020.1	Duck	Shantou	2006	H6N6
CY110022.1	Duck	Shantou	2006	H6N6
CY110024.1	Duck	Shantou	2006	H6N6
CY110032.1	Duck	Shantou	2006	H6N6
CY110040.1	Duck	Shantou	2006	H6N6
CY110042.1	Duck	Shantou	2006	H6N6
CY110044.1	Duck	Shantou	2006	H6N6
CY110046.1	Duck	Shantou	2006	H6N6
CY110048.1	Duck	Shantou	2006	H6N6
CY110050.1	Duck	Shantou	2006	H6N6
CY110172.1	Duck	Shantou	2006	H6N6
CY110204.1	Duck	Shantou	2006	H6N6
CY110244.1	Duck	Shantou	2006	H6N6
CY110252.1	Duck	Shantou	2006	H6N6
CY110310.1	Duck	Shantou	2006	H6N6
CY110314.1	Duck	Shantou	2006	H6N6
CY110352.1	Duck	Shantou	2006	H6N6
CY110366.1	Duck	Shantou	2006	H6N6
CY110372.1	Duck	Shantou	2006	H6N6
CY110386.1	Duck	Shantou	2006	H6N6
CY110392.1	Duck	Shantou	2006	H6N6
CY110394.1	Duck	Shantou	2006	H6N6
CY110408.1	Duck	Shantou	2006	H6N6
CY110416.1	Duck	Shantou	2006	H6N6
CY110424.1	Duck	Shantou	2006	H6N6
CY110432.1	Duck	Shantou	2006	H6N6
HM144708.1	Duck	Shantou	2001	H6N6
HM144711.1	Duck	Shantou	2004	H6N6
HM144712.1	Duck	Shantou	2005	H6N6
HM144715.1	Duck	Shantou	2005	H6N6
HM144716.1	Duck	Shantou	2005	H6N6
HM144720.1	Duck	Shantou	2005	H6N6
HM144719.1	Goose	Shantou	2005	H6N6

HM144714.1	Mallard	Shantou	2005	H6N6
CY109372.1	Wild Duck	Shantou	2006	H6N6
CY109436.1	Wild Duck	Shantou	2006	H6N6
HM144709.1	Wild Duck	Shantou	2004	H6N6
HM144713.1	Wild Duck	Shantou	2005	H6N6
AB304149.1	Duck	Shiga	2004	H4N6
AB292663.1	Duck	Siberia	1996	H4N6
AB295610.1	Duck	Siberia	2001	H4N6
CY076986.1	Dunlin	Sweden	2005	H4N6
CY060276.1	Mallard	Sweden	2002	H10N6
CY060352.1	Mallard	Sweden	2003	H4N6
CY121909.1	Mallard	Sweden	2005	mixed
CY121920.1	Mallard	Sweden	2005	mixed
AY207551.1	Duck	Taiwan	2000	H4N6
GU396864.1	Duck	Taiwan	2006	H4N6
GU396865.1	Duck	Taiwan	1999	H4N6
GU396866.1	Duck	Taiwan	2007	H4N6
GU396867.1	Duck	Taiwan	2007	H4N6
GU396868.1	Duck	Taiwan	2006	H4N6
GU396869.1	Duck	Taiwan	2006	H4N6
GU396870.1	Duck	Taiwan	2006	H4N6
GU396871.1	Duck	Taiwan	2006	H4N6
GU396872.1	Duck	Taiwan	2006	H4N6
GU396874.1	Duck	Taiwan	2006	H4N6
GU396875.1	Wild Duck	Taiwan	2006	H4N6
GU396876.1	Wild Duck	Taiwan	2007	H4N6
GU396877.1	Wild Duck	Taiwan	2001	H4N6
GU396878.1	Wild Duck	Taiwan	1998	H4N6
GU396879.1	Wild Duck	Taiwan	2000	H4N6
GU396880.1	Wild Duck	Taiwan	2002	H4N6
GU396881.1	Wild Duck	Taiwan	2004	H4N6
GU396882.1	Wild Duck	Taiwan	2005	H4N6
GU396883.1	Wild Duck	Taiwan	2005	H4N6
GU396884.1	Wild Duck	Taiwan	2006	H4N6
JX307117.1	Duck	Thailand	2010	H7N6
JX307119.1	Duck	Thailand	2010	H7N6
JX307129.1	Duck	Thailand	2010	H7N6
JX307144.1	Duck	Thailand	2010	H7N6
JX307149.1	Duck	Thailand	2010	H7N6
JX307151.1	Duck	Thailand	2010	H7N6
JX307156.1	Duck	Thailand	2010	H7N6
JX307172.1	Duck	Thailand	2010	H7N6
JX307177.1	Duck	Thailand	2010	H7N6
JX307181.1	Duck	Thailand	2010	H7N6
JX307186.1	Duck	Thailand	2010	H7N6
JX307191.1	Duck	Thailand	2010	H7N6
JX307198.1	Duck	Thailand	2010	H7N6

JX307215.1	Duck	Thailand	2010	H7N6
JX307220.1	Duck	Thailand	2010	H7N6
JX307225.1	Duck	Thailand	2010	H7N6
JX307227.1	Duck	Thailand	2010	H7N6
JX307242.1	Duck	Thailand	2010	H7N6
JX307252.1	Duck	Thailand	2010	H7N6
CY062550.1	Muscovy Duck	Thailand	2009	H4N6
CY062558.1	Muscovy Duck	Thailand	2009	H4N6
AB545604.1	Duck	Vietnam	2009	H4N6
AB719908.1	Duck	Vietnam	2011	H3N6
AB719940.1	Duck	Vietnam	2011	H4N6
AB728597.1	Duck	Vietnam	2012	H4N6
AB739023.1	Duck	Vietnam	2012	H3N6
AB739665.1	Duck	Vietnam	2012	H3N6
AB746476.1	Duck	Vietnam	2011	H4N6
EU880344.1	Mallard	Yan chen	2005	H4N6
CY109716.1	Duck	Yunnan	2007	H6N6
AB569521.1	Goose	Zambia	2008	H4N6
AB470298.1	Pelican	Zambia	2006	H3N6
FJ349248.1	Mallard	ZhaLong	2004	H4N6
